# A breakdown of metabolic reprogramming in microglia induced by CKLF1 exacerbates immune tolerance in ischemic stroke

**DOI:** 10.1186/s12974-023-02779-w

**Published:** 2023-04-25

**Authors:** Wen-yu Ma, Qing-lin Wu, Sha-sha Wang, Hong-yun Wang, Jun-rui Ye, Hong-shuo Sun, Zhong-ping Feng, Wen-bin He, Shi-feng Chu, Zhao Zhang, Nai-hong Chen

**Affiliations:** 1 Science and Technology Innovation Center, Guangzhou University of Chinese Medicine, Guangzhou, 510405 China; 2 State Key Laboratory of Bioactive Substances and Functions of Natural Medicines, Institute of Materia Medical & Neuroscience Center, Chinese Academy of Medical Sciences and Peking Union Medical College, Beijing, 100050 China; 3 Department of Physiology, Temerty faculty of Medicine, University of Toronto, 1 King’s College Circle, Toronto, ON M5S 1A8 Canada; 4 Shanxi Key Laboratory of Chinese Medicine Encephalopathy, Shanxi University of Chinese Medicine, Taiyuan, 030024 China; 5 National International Joint Research Center for Molecular Chinese Medicine, Shanxi University of Chinese Medicine, Taiyuan, 030024 China

**Keywords:** CKLF1, Microglia, Metabolic reprogramming, Immune tolerance, Ischemic stroke, Phagocytosis

## Abstract

Ischemic stroke is characterized by the presence of reactive microglia. However, its precise involvement in stroke etiology is still unknown. We used metabolic profiling and showed that chemokine like factor 1 (CKLF1) causes acute microglial inflammation and metabolic reprogramming from oxidative phosphorylation to glycolysis, which was reliant on the AMP-activated protein kinase (AMPK)–mammalian target of rapamycin (mTOR)–hypoxia inducible factor 1α (HIF-1α) signaling pathway. Once activated, microglia enter a chronic tolerant state as a result of widespread energy metabolism abnormalities, which reduces immunological responses, including cytokine release and phagocytosis. Metabolically dysfunctional microglia were also found in mice using genome-wide RNA sequencing after chronic administration of CKLF1, and there was a decrease in the inflammatory response. Finally, we showed that the loss of CKLF1 reversed the defective immune response of microglia, as indicated by the maintenance its phagocytosis to neutrophils, thereby mitigating the long-term outcomes of ischemic stroke. Overall, CKLF1 plays a crucial role in the relationship between microglial metabolic status and immune function in stroke, which prepares a potential therapeutic strategy for ischemic stroke.

**Graphical Abstract:**

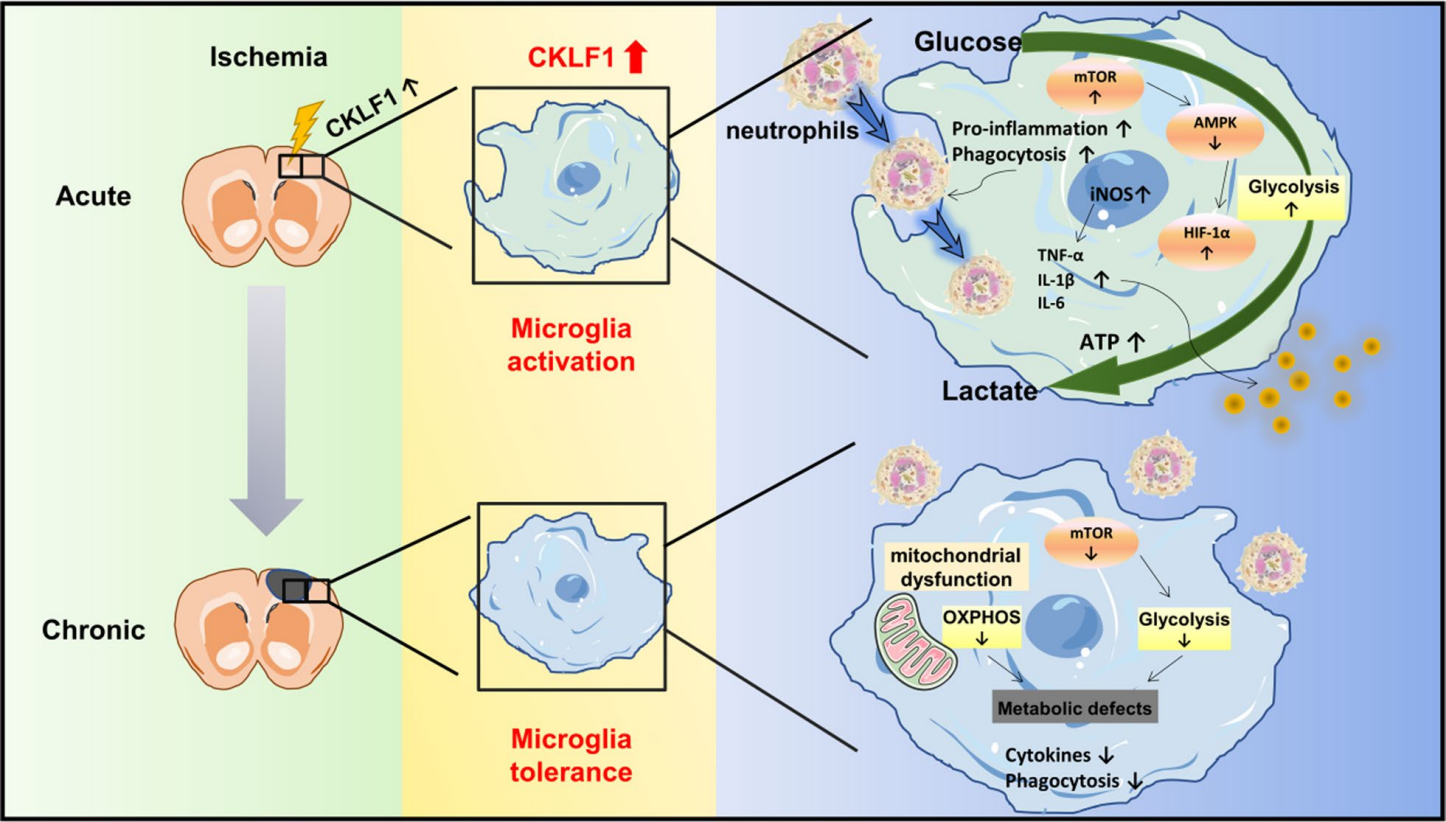

**Supplementary Information:**

The online version contains supplementary material available at 10.1186/s12974-023-02779-w.

## Introduction

Ischemic stroke is the most common type of stroke, and neurological injury has been associated with a variety of pathological indicators, including inflammation, oxidative stress, and blood–brain barrier breakdown. The innate immune response is a highly complex physiological process during the acute stage of ischemic stroke. Microglia, which are the brain's resident immune cells, are initially activated and have the most intricate effects during the post-stroke stage [1]. It has been revealed that activation of microglia is the primary driver of neuroinflammation, the loss of microglia exacerbates neuronal damage, and repopulating microglia following stroke promotes the alleviation of neurological damage significantly [2, 3], suggesting that the immune status of microglia is important for stroke outcomes. However, the regulatory mechanism remains unclear.

Microglia are polysynaptic and flexible immunological effector cells in the central nervous system, that may develop an inflammatory phenotype in response to "danger signals" such as pathogens and tissue damage, as evidenced by increased production of pro-inflammatory cytokines such as interleukin-1β (IL-1β), interleukin-6 (IL-6) and tumor necrosis factor-α (TNF-α), as well as enhanced immune responses, including cellular recruitment (e.g., chemotaxis, and phagocytosis) [4]. Consequently, activated microglia are attracted to the inflammatory region and facilitate the elimination of pathogens. However, once this process begins, it must be tightly monitored, as uncontrolled or severe inflammation can cause host tissue damage [5, 6]. Many pathways regulating these characteristics have been elucidated [7]. However, the distinction between homeostatic and disease-related signaling is not absolute [8], and this simplistic view does not adequately describe the complex physiology of microglia [9].

As a result of immune cells becoming polarized during inflammation, innate immunological tolerance occurs, which is a host-protective mechanism in which these cells become unresponsive to recurrent stimulation. Immune tolerance may protect host tissue from the detrimental effects of excessive inflammatory responses. However, it has been paradoxically associated with immune cell malfunction in a variety of disorders, including cancer, sepsis, autoimmune diseases, and metabolic diseases [10, 11], in vitro and in vivo. Therefore, more biomarkers and underlying mechanisms to reflect the functions of microglia in different stages needs to be investigated [12].

Recently, research on cellular metabolic reprogramming has provided new clues for the activation of immune cells. Because immune cells perform a variety of functions and have different energy demands, the metabolic pathways that are used to generate energy change developing on cellular phenotype. Relevant research has shown that the reprogramming of the metabolic state from oxidative phosphorylation (OXPHOS) to aerobic glycolysis is beneficial for immune cells to play a pro-inflammatory role which could provide cells with a rapid supply of adenosine triphosphate (ATP), thus regulating the enzyme and the production of pro-inflammatory cytokines [13]. Although glycolysis is less efficient than oxidative phosphorylation in terms of ATP production, the rate of ATP production by glycolysis is 10–100 times faster than that of OXPHOS, allowing glycolysis to mediate energy-intensive activities, such as proliferation, migration, cytokine release, and phagocytosis [14]. On the other hand, naïve and resting cells, which require a continuous energy source and rely mostly on mitochondrial respiration. As a result, microglia might adopt a phenotype known as trained immunity, in which immunological responses are reinforced by an increase in glycolysis [15–17]. Metabolic reprogramming results in the production of large number of pro-inflammatory factors to increase immune memory. A change in the cell metabolic state is also closely related to the formation of immune memory by macrophages [18]. However, it is still unclear whether metabolic reprogramming affects the immune function of microglia.

Recent clinical study showed machine learning algorithm applied to clinical cohort to predict chemokines as biomarkers for stroke outcomes in ischemic stroke patients, such as CCL4, CXCL3 were associated with infract volume and CCL3 was related with edema [19]. Chemokine-like factor 1 (CKLF1) is a CC chemokine that was cloned in 2001 and has multiple biological activities [20]. As a secreted protein [21, 22], CKLF1 plays a key role in a variety of tissues and has potential effects, which has attracted extensive attention from researchers [20]. Studies have shown that CKLF1 is highly expressed after cerebral ischemia [23, 24]. In the acute phase following stroke, knocking out (KO) CKLF1 or inhibiting its activity might cause severe nerve injury and decrease the number of activated microglia. A single dose of CKLF1 administered 1 h prior to ischemia can exacerbate nerve damage and the inflammatory response, suggesting that CKLF1 can be used to immunologically train microglia and then rapidly activate microglia in the presence of subsequent ischemic injury [25, 26]. However, it is unknown whether CKLF1 can induce immunological tolerance or mitigate nerve damage following stroke, and the regulation of CKLF1 expression and its molecular mechanism associated with changes in microglial phenotypic changes have not yet been elucidated, which seriously hinders research on this chemokine as a new anti-stroke therapeutic target.

Hence, the core aim of this study is to investigate whether CKLF1 has a time-dependent activation microglia, whether these changes depend on the regulation of energy metabolism. To address the above aims, we quantified the effects of CKLF1 on microglial function by phagocytosis and cytokines production, as well as the energy metabolism. It was found that CKLF1 causes acute microglial inflammation and metabolic reprogramming from oxidative phosphorylation to glycolysis, once activated, they entered a chronic tolerant state as a result of widespread energy metabolism abnormalities and therefore reduced immunological responses. The loss of microglial immunological response exacerbated outcomes of stroke, which could be reversed by knocking out or neutralization of CKLF1, which providing a new clue for the treatment of ischemic stroke.

## Materials and methods

### Animals

All animal procedures were performed according to protocols approved by the Animal Care and Use Committee of the Peking Union Medical College and the Chinese Academy of Medical Sciences. Adult male (7–8 weeks old) C57BL/6N mice (Charles Rivers, China) were housed with a constant temperature and a 12:12 h light/dark cycle. Food and water were consumed ad libitum by the mice. CKLF1^−/−^ mice were generously provided by Professor Zhang (Institute of Laboratory Animal Science, Peking Union Medicine College, Chinese Academy of Medical Sciences)[25]. CKLF1^−/−^ mice were generated using the CRISPR/Cas9 system. Investigators designed sgRNA-target sequences (gene ID: 75458, target site 1: cctggagcagcgtttgctcgg, target site 2: gatattatacttgtaatctgg) based on the first coding exon of the CKLF1 gene and transcribed sgRNA and Cas9 mRNA in vitro. After injecting into fertilized eggs, investigators obtained 23 pups in the F0 generation, two of which were KO mice, as determined by the test of PCR and sequencing. After being mated for 10 generations, investigators obtained homozygous CKLF1^−/−^ mice with genetic stability. In this line, a 957-bp nucleic acid in CKLF1 was deleted, and a frameshift mutation occurred in the subsequent protein coding region. After adaptive feeding, all animals were assigned to the control group and the experimental group, strictly following the randomization and blinding procedures.

### Stereotaxic injection and photothrombotic stroke

Surgery was performed under isoflurane anesthesia, and the mice were fixed using a stereotactic frame (RWD Life Science, China). The injection site was the right M1 area according to the second edition of The Mouse Brain with stereotaxic coordinates from the bregma point as follows: AP, + 1.5 mm; ML, − 2.0 mm; DV, -1.7 mm. After the injection, the needle was maintained in the M1 area for the next 10 min to prevent drug leakage. For the administration cannula, surgery was performed with brain stereotaxic apparatus. After the animals were anesthetized, the scalp was opened to expose the skull, and the site was drilled. The cannula was clamped by a holder and implanted to a suitable depth. The dental cement was fixed and the catheter cap was inserted to complete the surgery. For acute stimulation of CKLF1, 24 h after stereotactic injection of C27 dissolved in PBS at the dose of 10 μg, which is the c-terminal peptide of CKLF1 [27] (sequenced: ALIYRKLLFNPSGPYQKKPVHEKKEVL; 99% purity; Guoping Pharmaceutical Anhui, China), the brains were collected after transcardial perfusion. For tolerated stimulation, 10 μg of C27 was administered once per day for four days in the same manner. Twenty-four hours after the last injection, the brains were collected following transcardial perfusion. For anti-CKLF1 antibody treatment, 10 μg of anti-CKLF1 or control IgG was administered by stereotactic injection prior to photothrombotic stroke (PT). After that, 200 μL of 10 mg/mL Rose Bengal sodium salt (Solarbio, China) solution was administered via the tail vein. After 5 min, a green laser with a spot diameter of 2 mm (wavelength 532 nm) was used to irradiate the animals for 7 min to form an ischemia model in specific brain regions.

### Primary microglia culture

Primary microglia (PMG) were obtained in the cerebral cortex of C57BL/6 neonatal mice 24 h after birth. The decapitated mice were placed in precooled DMEM/F12 medium. Brain tissue was cut into pieces to digest the cells and then sieved, and the single-cell suspension was placed in culture flasks coated with poly-lysine (PLL). After 3 days of incubation at 37 °C and 5% CO_2_, the medium (DMEM/F12 containing 10% fetal bovine serum and 1% penicillin–streptomycin) was changed. Microglia were isolated from the mixed glial cultures on Day 10 by oscillation, and shaking was repeated 3–5 days later. Isolated PMG were seeded at a density of 1.5–2.0 × 10^5^ cells/mL on a PLL-coated cell culture plate and incubated until stable (37 °C, 5% CO_2_).

### Stimulation of PMG

PMG were stimulated with LPS (100 ng/mL) and IFN-γ (20 ng/mL) or C27 (500 nM, 1000 nM, 2000 nM) for 24 h. In some experiments, PMG were preincubated with 2-DG (4 mM, Topscience, China) to inhibit glycolysis or with rapamycin (50 nM, Topscience, China) or metformin (2 mM, Topscience, China) to block the mTOR pathway. Experiments on the tolerance model mimicked chronic conditions by incubating cells with vehicle or C27 (1000 nM) for 24 h, washing the cells with preheated phosphate buffered saline (PBS), and further incubating the cells for 3 days. After that, the cells were restimulated with C27 (1000 nM) for 24 h.

### Measurement of lactate, pyruvate kinase, pyruvate dehydrogenase and glucose-6-phosphate

PMG (2.0 × 10^5^ cells/mL) were seeded on a 6-well plate and treated as described above. Then, lactic acid and pyruvate kinase levels in the cells were quantified with the lactic acid content assay kit (Solarbio, China), pyruvate kinase activity detection kit (Solarbio, China) and Pyruvate dehydrogenase (PDH) activity detection kit (Solarbio, China) according to the manufacturer's instructions. PMG (1 × 10^6^) were homogenized in triplicate ice-cold PBS. Samples were transferred to 10-kDa molecular weight cutoff spin filters (YM-10 from Millipore, USA) for deproteinization. The samples were centrifuged at 13,000 g for 10 min to remove the insoluble material. Add 50 μL of sample and standard to 96-well plates using a glucose-6-phosphate (G6P) kit (MAK014, Sigma, USA) and follow the kit instructions, measure the absorbance at 450 nm.

### Enzyme-linked immunosorbent assay (ELISA)

The levels of IL-1β, TNF-α, IL-10 (Fankewei, China) in tissues were measured by ELISA technique according to the manufacturer's instructions.

### Live cell mitochondrial imaging

PMG were cultured on fluorodish cell culture dishes (World Precision Instruments, China) to image live mitochondria and were treated with MitoTracker Green (300 nM, Thermo Fisher Scientific, USA) solution in the dark for 30 min (37 °C, 5% CO_2_) to label mitochondria. Fresh complete medium was added after the cells were rinsed with preheated PBS. Real-time mitochondrial imaging was performed using confocal laser scanning microscopy (Leica, Germany) and mitochondrial morphology was analyzed by using ImageJ software [28].

### Western blotting

Cultured cells were collected and then lysed with RIPA lysis buffer (Beyotime, China) containing a mixture of protease and phosphate inhibitors. Proteins were separated by electrophoresis on a 10% SDS-PAGE gel containing equal amounts of protein (25–60 μg) lysates and transferred to a PVDF (Merck Millipore, USA) membrane. After 2 h of blocking with 5% bovine serum albumin (BSA) in Tris-buffered saline containing 0.1% Tween-20 (TBS-T) at room temperature (RT), the membranes were incubated overnight with the following primary antibodies at 4°℃: anti-IL-6 (1: 1000, Abcam, ab208113, UK), anti-mTOR [Y391] (1:1000, Abcam, ab32028, UK), anti-mTOR (phospho S2481) (1:1000, Abcam, ab137133, UK), anti-AMPK alpha 1 + AMPK alpha 2 (1:1000, Abcam, ab131512, UK), anti-AMPK alpha 1 (phospho T183) + AMPK alpha 2 (phospho T172) (1:1000, Abcam, ab133448, UK), anti-TREM2 (1:500, Abcam, ab86491, UK), anti-HIF-1α (1:1000, Abcam, ab113642, UK), anti-β-actin (1:10,000, ABclonal, AC026). After 3 washes, the specific blot was incubated with secondary antibodies against the appropriate species at RT for 2 h. The expression of each protein was examined with an enhanced chemiluminescence plus detection system (Molecular Device, Lmax). Analysis was performed by FIJI software, and the data were presented as normalized to control in each experiments.

### Isolation of RNA and quantitative real-time polymerase chain reaction (RT-PCR)

Total RNA was extracted from isolated microglia by using TRIzol reagent (Invitrogen, Carlsbad, CA, USA), and dissolved in 20 μL of DEPC (Beyotime, China) to obtain total RNA. The RNA was quantified by measuring the OD at 260 and 280 nm using a NanoDrop 2000 spectrophotometer and stored at -80℃. The synthesis of cDNA was performed according to the instructions of the reverse transcription cDNA synthesis kit (Transgen, China). In brief, 2 μg of total RNA was incubated for 15 min at 42°℃ and for 5 s at 85°℃ to obtain cDNA, which was diluted with DEPC water and stored at − 20°℃. qPCR System (Foster City, CA, USA) using the TransStart Tip Green qPCR Supermix kit (TransGen, China). The PCR amplification conditions were as follows: 94 °C for 30 s for pre-denaturation, followed by 94 °C for 5 s and 60 °C for 30 s for denaturation for 40 cycles for extension. The primers are as follows:

Inducible nitric oxide synthase (iNOS), forward primer: 5’-CAAGCACCTTGGAAGAGGAG-3’ and reverse primer: 5’-AAGGCCAAACACAGCATACC-3’.

Fc gamma receptor type III (CD16),forward primer: 5’-TTTGGACACCCAGATGTTTCAG-3’ and reverse primer: 5’-GTCTTCCTTGAGCACCTGGATC-3’.

Fc gamma receptor type II (CD32),forward primer: 5’-AATCCTGCCGTTCCTACTGATC-3’ and reverse primer: 5’-GTGTCACCGTGTCTTCCTTGAG-3’.

IL-6, forward primer: 5’-GGAGCCCACCAAGAACGATA-3’ and reverse primer: 5’-TCACCAGCATCAGTCCCAAG-3’.

IL-1β,forward primer: 5’-GCCCATCCTCTGTGACTCAT-3’ and reverse primer: 5’-AGCTCATATGGGTCCGACAG-3’.

TNF-α,forward primer: 5’-AGAAGTTCCCAAATGGCCTC-3’ and reverse primer: 5’-CCACTTGGTGGTTTGCTACG-3’.

Arginase-1 (Arg-1),forward primer: 5’-CTCCAAGCCAAAGTCCTTAGAG-3’ and reverse primer: 5’-AGGAGCTGTCATTAGGGACATC-3’.

Chemokine (CC motif) ligand-22 (CCL-22),forward primer: 5’-CTGATGCAGGTCCCTATGGT-3’ and reverse primer: 5’-GCAGGATTTTGAGGTCCAGA-3’.

Transforming growth factor β (TGF-β),forward primer: 5’-TGCGCTTGCAGAGATTAAAA-3’ and reverse primer: 5’-CGTCAAAAGACAGCCACTCA-3’.

Pyruvate kinase M2 (PKM2),forward primer: 5’-AGTACGCCCGAGGACTTC-3’ and reverse primer: 5’-AAATGATGCCAGTGTTGCGG-3’.

Hexokinase (HK), forward primer: 5’-GCTGGAGTCTTGTCAGGCATT-3’ and reverse primer: 5’-AATCCAAGCGCGAAACCAAA-3’.

6-phosphofructo-2-kinase/fructose-2,6-bisphosphatase 3 (PFKFB3), forward primer: CGGGAGAGGTCAGAGAACATGAA-3’ and reverse primer: 5’-CTTCAACATGCCGACCTCCA-3’.

Glucose transporter type 1 (GLUT1),forward primer: 5’-AGCAGAGGCTTGCTTGTAGAG-3’ and reverse primer: 5’-GCCCGTCACCTTCTTGCT-3’.

Mitochondrial pyruvate carrier 1 (MPC1), forward primer: 5'-ATGAGTACGCACTTCTGGGG-3' and reverse primer: 5'-CGCCCACTGATAATCTCTGGA-3'.

Mitochondrial pyruvate carrier 2 (MPC2), forward primer: 5'-TACCACCGACTCATGGATAAAGT-3' and reverse primer: 5'-CACACACCAATCCCCATTTCA-3'.β-actin,forward primer: 5’-CATCCGTAAAGACCTCTATGCCAAC-3’ and reverse primer: 5’-ATGGAGCCACCGATCCACA-3’.

To interpret the results, Ct value data from the reactions were collected using the corrected threshold setting. β-actin was used as an endogenous control, and the fold changes were normalized to the control group. The 2-ΔΔCt method was used for relative quantification.

### Measurement of real-time extracellular acidification rate (ECAR) and oxygen consumption rate (OCR)

The ECAR and OCR were estimated using a Seahorse XF24 analyzer (Agilent, USA). A total of 4 × 10^4^ cells were plated in XF24 cell culture microplates and cultured as instructed and stimulated by drug administration. The plates were hydrated and incubated overnight (37 °C, no carbon dioxide) with XF calibration buffer; the assay medium (XF minimal medium containing 1 mM pyruvic acid, 4 mM glutamine and 25 mM glucose) was added prior to the assay. For ECAR measurement, after the cell plate and the probe plate were loaded with and stabilized by the XF analyzer, glucose, oligomycin, and 2-DG were successively administered to the cells, and then the change in the ECAR was monitored in real time. After the ECAR experiment, the key parameters of glycolytic flux were calculated, including basic glycolysis, maximum glycolysis capacity, and glycolysis reserve and nonglycolysis acidification. For OCR measurement, the mixture of oligomycin, FCCP and rotenone and antimycin A was sequentially injected to monitor the changes in the OCR in real time [29], and each well on the cell culture plate was quantified, followed by normalization of the data. Basal respiration, ATP production, maximal respiration, spare capacity, and proton leak were calculated.

### Microparticle uptake assay

Cells (5 × 10^5^/mL) were seeded in a 6-cm dish and cultured for 24 h until the cells adhered. The negative control, treatment control and each medication administration groups were set, and the cells were stimulated for 24 h. Then, 0.75 mg (1.0 × 10^7^ Zymosan A particles) of microsphere particles (Zymosan A (S. Cerevisiae) Bioparticles, Alexa Fluor™ 488 conjugate) were coated by incubation with 1.5 mL of DMEM containing 50% FBS for 15 min with shaking at 37 °C (1000 rpm) and tenfold dilution in preheated DMEM containing 10% FBS. The prepared microspheres (1 × 10^6^ /mL) were added and incubated with the cells at 37 °C for 120 min (the negative control had no particles). The cells were washed with PBS, blown off with complete medium, and transferred to an EP tube for centrifugation at 1000 rpm for 4 min, and the supernatant was discarded. The cells were re-suspended for a second time with PBS containing 2% FBS, blown evenly, passed through a 70-μm sieve, and centrifuged at 1000 rpm for 4 min. A 50 μL cell suspension was discarded and retained in a 1.5-mL EP tube. The phagocytic rate of the cells was then detected by ImageStream system flow cytometry (Merck, France). The results were analyzed by using IDEAS 6.2 software.

### Lactate dehydrogenase (LDH) and terminal deoxynucleotidyl transferase dUTP nick end labeling (TUNEL) assay

To quantify the cytotoxicity of the different stimulations, LDH was measured using the LDH cytotoxicity assay kit (Zhongshengbeikong Biotechnology, China) according to the manufacturer's instructions. Microglia (4 × 10^4^/well) were inoculated on 96-well plates and treated as described. Medium containing the released LDH was transferred into EP tubes, and LDH activity was measured using a biochemical analyzer.

To quantify apoptosis under experimental conditions, microglia (4 × 10^4^/well) were seeded on 96-well plates, and TUNEL (Beyotime, China) staining was performed according to the manufacturer's instructions with DNaseI (100 U/mL) treated cells as the positive control. Images were observed with a fluorescence microscope and analyzed using ImageJ software.

### Microglial isolation from adult mouse brains

For the sorting experiment, the in vivo acute stimulation model was constructed by brain-targeted injection of C27 for 24 h, and the in vivo immune tolerance model was constructed by brain-targeted injection of PBS or C27 for four consecutive days, after which microglia were isolated from the mouse brain. In short, the cells were incubated with precooled Dulbecco's PBS (dPBS; Sigma-Aldrich, USA), and the brain (excluding the olfactory bulb and cerebellum) was transferred to a solution containing an enzyme mix (Miltenyi Biotec, Germany). The tissue was cut into pieces and enzymatically digested using a gentleMACS Dissociator Homogenizer (Miltenyi Biotec, Germany) running the program Brain-01-03. The cell solutions were passed through a 70-μm cell filter (Falcon) to obtain a single-cell suspension and debris was removed using a fragment removal solution (Miltenyi Biotec, Germany). CD11b (microglia) microbeads (Miltenyi Biotec, Germany) and magnetically activated cell sorting (MACS; Miltenyi Biotec, Germany) system were used. The isolated microglia were lysed with precooled TRIzol reagent and stored at -80 °C.

### RNA sequencing of microglia

Total RNA was extracted from the isolated microglia and subjected to RNA quality testing, and messenger RNA was purified by mRNA-specific polyA structure using the Truseq Stranded mRNA Lt Sample Prep kit. First strand cDNA was synthesized by random primers and reverse transcriptase, and second strand cDNA was synthesized using the first strand cDNA as a template. After purifying the double-stranded cDNA and performing terminal repair, an A was added at the 3’ end, a sequencing linker was added by the ligase, and performing fragment selection on the product with the linker added by a magnetic bead mode. The pooled libraries were uniformly diluted to 2 nM and denatured with base to form a single-stranded library. Sequencing was performed on the Novaseq 6000 PE150 platform. Filtering was performed to obtain high-quality clean data by comparing the clean data with a designated reference genome, calculating the comparison efficiency of the sequencing data with the reference genome, and evaluating the saturation of the sequencing data and the gene coverage. Differential genes were screened in different sample groups, and visualized displays such as clustering analysis and volcanic diagrams were conducted on the differential genes. GO/KEGG functional annotation and functional enrichment analysis were conducted on the differential genes to examine the functions and regulatory relationships of the differential genes.

### Immunohistochemistry and immunofluorescence analysis

For immunofluorescence analysis, the mice were anesthetized and perfused with cold PBS and the brains were incubated for 24 h in 4% PFA and then in 30% sucrose paraformaldehyde for full dehydration. Serial coronal Sects. (30 μm thick) of the brain were prepared using a cryomicrotome (Leica, Germany). The sections were immersed sequentially in antigen fixation solution for 15 min, in 1% Triton X-100 for 10 min and in 5% BSA for 30 min and then incubated overnight at 4℃ with ionized calcium binding adapter molecule 1 (Iba-1) primary antibodies (1: 1000, Wako Pure Chemical Industries, Japan). For cell immunofluorescence analysis, cells were inoculated at a density of 1.5 × 10^5^/mL. The cells were cultured at 37 °C with 5% CO_2_ until they reached 70% confluence and were then stimulated with C27 and the positive control LPS + IFN-γ. After 24 h, immunofluorescence analysis was performed. After the cells were washed, the cells were fixed in 4% paraformaldehyde 20 min. The sections were washed with PBS, and the membrane was broken using 0.3% Triton X-100 for 10 min. The surface of the sections was covered with 3% BSA and blocked at RT for 1 h. The primary antibodies were added and incubated overnight at 4 °C. The next day, the corresponding Alexa 488 or 546-coupled IgG secondary antibodies (Thermo Fisher Scientific, USA) were added and incubated in the dark at RT for 1 h. Then, the cells were washed in PBS for 5 min. Hoechst was added, and the samples were covered with a cover glass, protected from light. After the slices were sealed, they were observed under a confocal laser scanning microscope (Citation 10, Biotech, USA) and the acquired images were analyzed by ImageJ software.

### Behavioral assessment

#### Cylinder test

The mice were placed in a cylindric transparent plexiglass chamber (D × H: 15 cm × 20 cm) and allowed to move freely. The process was recorded, and a mirror was mounted behind the cylinder to capture the mice when they turned their back to the camera. Each upper limb contact with the cylinder wall in an upright position until the end (marked by the return of both upper limbs to the ground) was counted as a set of movements, and the first 20 groups were recorded. Their performance was evaluated by counting the number of impaired forelimb contacts, which was calculated as a percentage of the total contacts.

#### Grid-walk test

Forelimb motor function was evaluated by counting the number of foot errors in the grid-walk test. The mice were placed on a square grid with a 3 cm × 3 cm mesh at a vertical height of 60 cm and were subjected to noise or other stimulation to traverse the mesh surface for 1 min. It was counted as a misstep when the mice inaccurately placed a limb and fell from the grid. The number of missteps on the left forelimb and the total number of uses of both forelimbs were calculated. The test was performed three times with an interval of 1 min. Injury analysis: number of missteps on the left forelimb (injured side) /total number of uses of both forelimbs.

#### Pole climbing test

The limb coordination ability and adhesion ability of the mice in the pole test were evaluated by measuring the rod climbing time. The device consisted of a piece of wood 50 cm long with a diameter of 1 cm that was wrapped with gauze to prevent the animals from slipping, and the bottom was placed in a feeding cage and covered with padding to prevent mice from being injured. A wooden ball was attached to the top of the stick to help the mouse stay on top of the stick. The time that the mouse crawled from the top of the stick to the bottom was recorded. There were three consecutive measurements, and the interval between each measurement was at least 30 min to ensure physical recovery. The average value of the three measurements was used for data analysis.

### Quantification and statistical analysis

Statistical analyses were performed using IBM SPSS Statistics 19.0 (IBM Corp., Armonk, NY, USA) and GraphPad Prism 7.0 (GraphPad Software La Jolla, CA, USA). All values are presented as the mean ± SD. Statistical significance between multiple groups were determined with one-way ANOVA, Dunnett's multiple comparisons test; two-way ANOVA, Tukey's multiple comparisons test. A value of *p* < 0.05 was considered statistically significant.

## Results

### Acute microglial inflammation induced by CKLF1 accompanied by metabolic reprogramming to glycolysis

**Fig. 1:**
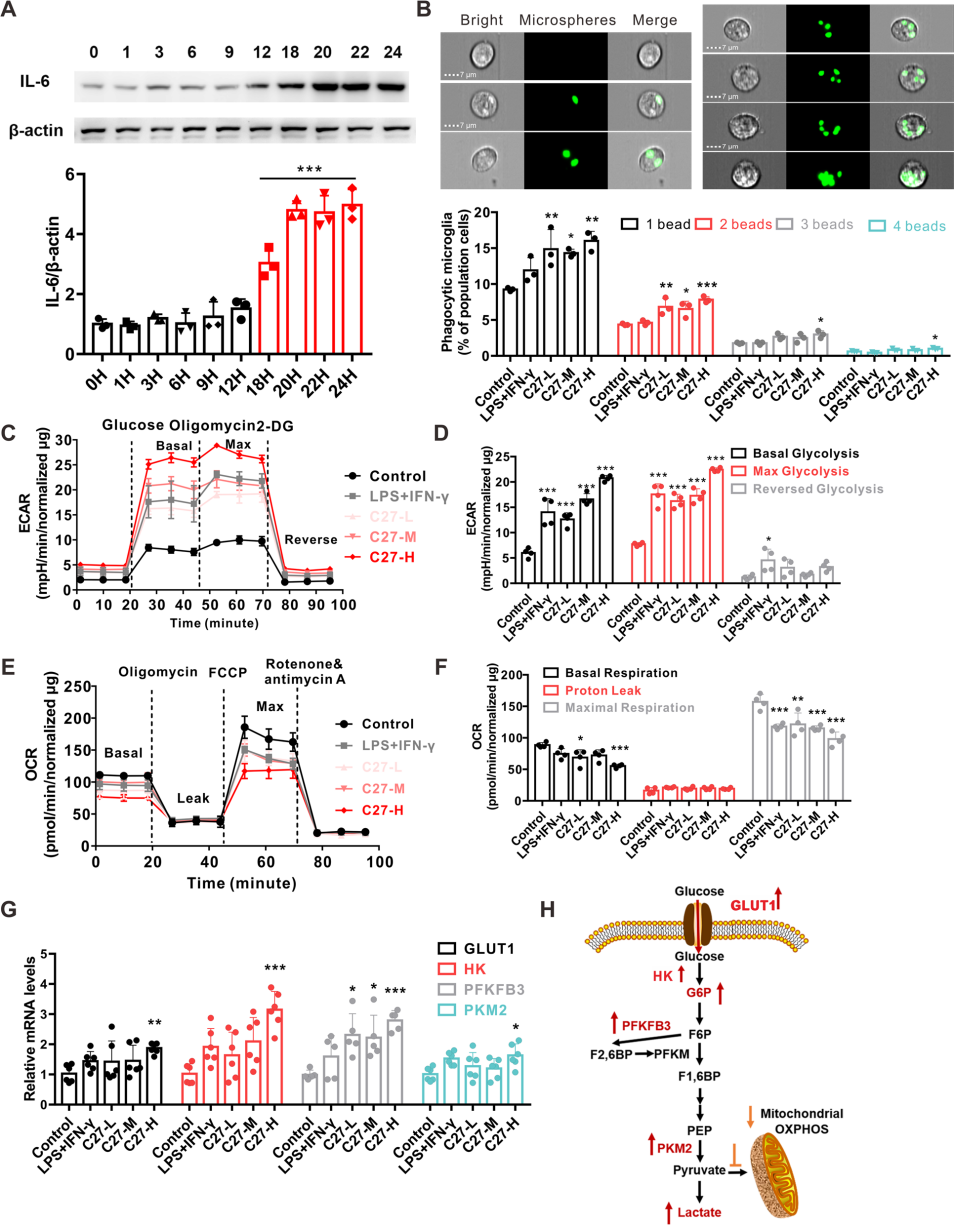
CKLF1 activates microglia and induces metabolic reprogramming to aerobic glycolysis. **A** Time course of IL-6 protein expression in response to C27 stimulation (*n* = 3 per group). *F* (9, 20) = 63.38. **B** Microglial phagocytosis was activated by C27 exposure. Microglial phagocytosis was tested using imaging and flow cytometry in the control group, positive control group (LPS + IFN-γ) and low-dose C27-L (500 nM), medium-dose C27-M (1000 nM), high-dose C27-H (2000 nM) administration groups (C27-L, C27-M, C27-H). The amount of zymosan that was phagocytosed by microglia was determined by fluorescence imaging of single cells. Scale bar: 7 μm. The proportion of zymosan phagocytosed by a single cell in each group (*n* = 3 per group). 1 bead: *F* (4, 10) = 8.54; 2 beads: *F* (4, 10) = 11.59; 3 beads: F (4, 10) = 5.078; 4 beads: *F* (4, 10) = 9.009. **C**, **D** Microglia glycolysis was enhanced by C27. The cell energy metabolism analyzer can detect real-time changes in extracellular acidification and quantitatively determine basic glycolytic capacity, maximum glycolytic capacity and reserve glycolytic capacity to measure the ECAR (*n* = 4 per group). Basal glycolysis: *F* (4, 15) = 51.77; Max glycolysis: *F* (4, 15) = 68.4; Reversed glycolysis: *F* (4, 15) = 3.952. **E** The cellular energy metabolism analyzer detects the real-time changes in the oxygen consumption rate of microglia (*n* = 4 per group). **F** Quantitative statistics of the basal respiration, proton leak and maximum oxygen consumption rate (*n* = 4 per group). Basal respiration: *F* (4, 15) = 9.005; Proton leak: *F* (4, 15) = 1.674; Maximal respiration: *F* (4, 15) = 14.82. **G** Relative mRNA expression of glycolytic genes stimulated by C27 (*n* = 5–6 per group). GLUT1: *F* (4, 25) = 2.787; HK: *F* (4, 25) = 8.762; PFKFB3: *F* (4, 20) = 7.334; PKM2: *F* (4, 25) = 3.341. **H** Schematic diagram of the metabolic reprogramming process induced by C27. The data are presented as the mean ± SD. *p < 0.05, **p < 0.01, ***p < 0.001 vs. control group

Primary microglia (PMG) from fetal mouse brain were cultured to examine their response to CKLF1. After 14 days of culture, high-purity PMGs were obtained (Additional file 1: Fig. S1A). PMG were treated with varying concentrations of CKLF1 peptide (C27) for different times. The levels of the inflammatory cytokine IL-6 were determined by western blotting. The expression of the inflammatory cytokine IL-6 was upregulated after CKLF1 treatment, gradually increased at 12 h, and peaked at 20–24 h (Fig. 1A). Next, we incubated microglia with different concentrations of CKLF1 for 24 h and used lipopolysaccharide and interferon γ (LPS + IFN-γ) as positive controls. It was found that the mRNA level of IL-6 was significantly upregulated by CKLF1 exposure (Additional file 1: Fig. S1B), and there is a concentration-dependent effect relationship of 500 nM to 2000 nM. The increase in IL-6 induced by CKLF1 was not significantly different from that of the positive control.

The increase in cytokines indicated that CKLF1 led to the activation of microglia. qPCR analysis showed that exposure to CKLF1 significantly increased the levels of CD16, CD32 and iNOS, which are marker genes of M1 polarization. M2 marker genes were examined, and CKLF1 inhibited the expression of CCL-22, but had no effect on TGF-β or Arg-1 (Additional file 1: Fig. S1D, E). These results suggest that CKLF1 induces an acute inflammatory response in microglia, reflecting recognition of CKLF1 is a danger signal to microglia.

Phagocytosis is the first step of immune cell defense. Normal microglial phagocytosis plays an important role in maintaining the brain homeostasis, brain development, pathological processes and brain regeneration [30, 31]. Therefore, we used imaging and flow cytometry to examine the ability of microglia to phagocytose microspheres to assess the effect of CKLF1 on microglial physiological functions. Our results showed that CKLF1 significantly enhanced phagocytosis (Additional file 1: Fig. S1C). Single-cell imaging showed that, CKLF1 increased the proportion of microglia with phagocytic functions, and significantly increased the proportion of cells that phagocytosed 1, 2, 3, and 4 globules, which suggested that phagocytic ability of microglia was improved by CKLF1 (Fig. 1B). Apoptosis was examined by TUNEL staining (Additional file 1: Fig. S1F), and necrosis was examined by LDH assays (Additional file 1: Fig. S1G). The results showed that the CKLF1-mediated enhancement of microglial activation and the inflammatory response was not related to apoptosis or necrosis.

Based on the close relationship between microglial state and energy metabolism, we monitored the metabolic dynamics of microglia by measuring the ECAR in real time with a Seahorse xFe24. The results showed that CKLF1 could significantly enhance the basal and maximal glycolytic capacity of microglia, which was characterized as a rapid increase in ECAR after the addition of a saturated concentration of glucose or the inhibition of ATP synthase activity with application of oligomycin. 2-Deoxy-D-glucose (2-DG) was the last drug added, which inhibited glycolysis by competitively binding to glycolytic pathway factor hexokinase, causing a decrease in ECAR, thus confirming that the ECAR in the experiment was derived from the glycolytic pathway (Fig. 1C, D) [32]. Elevated glycolysis was further evidenced by the enhancement of lactate production and pyruvate kinase activity (Additional file 2: Fig. S2A, B). Furthermore, 1000 nM CKLF1 significantly increased the expression of genes in the glycolytic pathway, including GLUT1, HK, PFKFB3, PKM2 (Fig. 1G) and G6P (Additional file 2: Fig. S2C). Moreover, MPC1 and MPC2, which are responsible for pyruvate transport and dehydrogenase, which are important indicators of mitochondria were downregulated following CKLF1 exposure (Additional file 2: Fig. S2D). These results indicated that CKLF1 enhanced the glycolytic capacity of microglia (Fig. 1H).

In addition to glycolysis, we found that CKLF1 significantly reduced basal OXPHOS levels and the maximal OXPHOS capacity in microglia by measuring changes in the OCR of microglia in real time (Fig. 1E, F). The decreased OCR value was found in the basal and maximal respiration, suggested that CKLF1 disrupted microglial mitochondrial function. Furthermore, we used MitoTracker Green to determine the form factor and aspect ratio of mitochondrial morphology in living cells (Additional file 2: Fig. S2F, G). The results showed that exposure to CKLF1-induced mitochondrial fission, which may be related to disruption of the electron transport chain [33]. These data further confirmed that microglial metabolism switched from OXPHOS to aerobic glycolysis during CKLF1-induced microglial activation.

### Inhibiting the glycolytic pathway blocks CKLF1-induced microglial activation

**Fig. 2:**
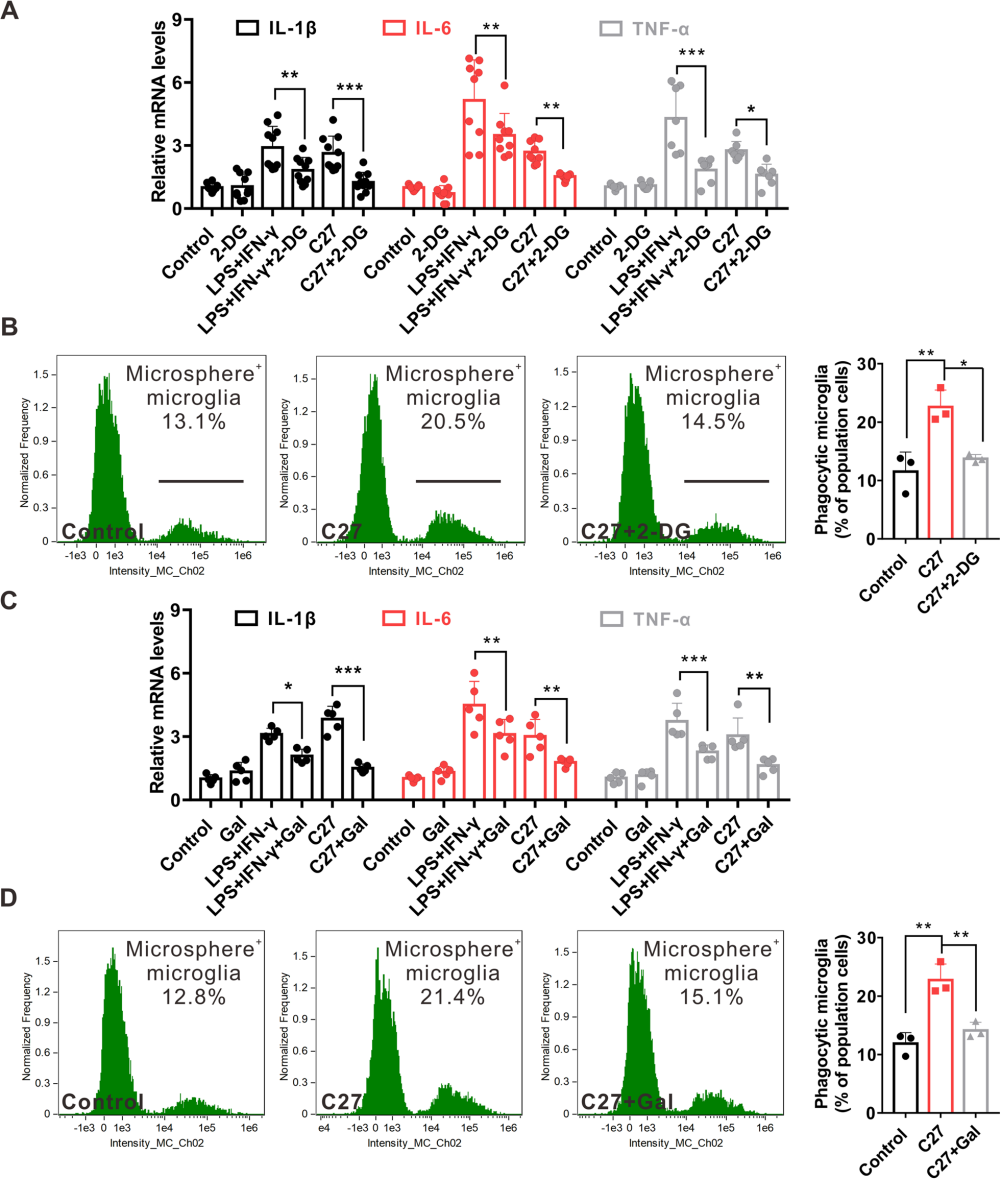
Inhibition of the glycolytic pathway abolished the activation of microglia induced by CKLF1. **A** Relative mRNA levels of inflammatory cytokines in microglia challenged by C27 with or without 2-DG (*n* = 8–10 per group). IL-1β: *F* (5, 54) = 15.51; IL-6: *F* (5, 48) = 29.78; TNF-α: *F* (5, 36) = 19.28. **B** Inhibiting of glycolysis blocked the phagocytosis induced by C27 in vitro. The amount of zymosan phagocytosed by microglia in each group was detected by imaging and flow cytometry, and the positive ratio of phagocytosis was quantified for each group (*n* = 3 per group). *F* (2, 6) = 15.43 **C** Relative mRNA levels of inflammatory cytokines in microglia challenged by C27 with or without galactose (*n* = 5 per group). IL-1β: *F* (5, 24) = 44.74; IL-6: *F* (5, 24) = 20.32; TNF-α: *F* (5, 24) = 19.1.** D** Inhibiting of glycolysis by galactose blocked the phagocytosis induced by C27 in vitro. The amount of zymosan phagocytosed by microglia in each group was detected by imaging and flow cytometry, and the positive ratio of phagocytosis was quantified for each group (*n* = 3 per group). *F* (2, 6) = 22.7. The data are presented as the mean ± SD. *p < 0.05, **p < 0.01, ***p < 0.001 vs. control group

To observe the role of glycolysis in CKLF1-induced microglial activation and the acute inflammatory response, we blocked glycolysis with 2-DG, which competitively binds to hexokinase to inhibit the glycolytic pathway. As mentioned previously, in the ECAR assay, 2-DG could offset the difference in ECAR elevation induced by different concentrations of CKLF1, suggesting that the functional status of microglia may also undergo corresponding changes. To test our hypothesis, we observed the effects of glycolysis on microglial function in vitro. qPCR analysis showed that the increase in cytokines induced by CKLF1 could be blocked by 2-DG, (Fig. 2A), and a microparticle-uptake assay showed that the CKLF1-induced increase in phagocytosis was significantly counteracted by 2-DG (Fig. 2B).

To further test if 2-DG had effects other than glycolysis inhibition, we used galactose (Gal), which must be metabolized by the Leloir pathway before entering glycolysis, leading to profound reduction in glycolytic flux and functionally effective inhibitory slow down [34]. Herein, we likewise found that the increase in cytokines induced by CKLF1 could be blocked by Gal (Fig. 2C). Moreover, a microparticle-uptake assay showed that the CKLF1-induced increase in phagocytosis was significantly counteracted by Gal (Fig. 2D), which showed that inhibiting the glycolytic pathway could significantly abolish the acute activation of microglia induced by CKLF1.

### CKLF1-induced glycolysis and inflammation are dependent on the AMPK–mTOR–HIF-1α pathway

**Fig. 3:**
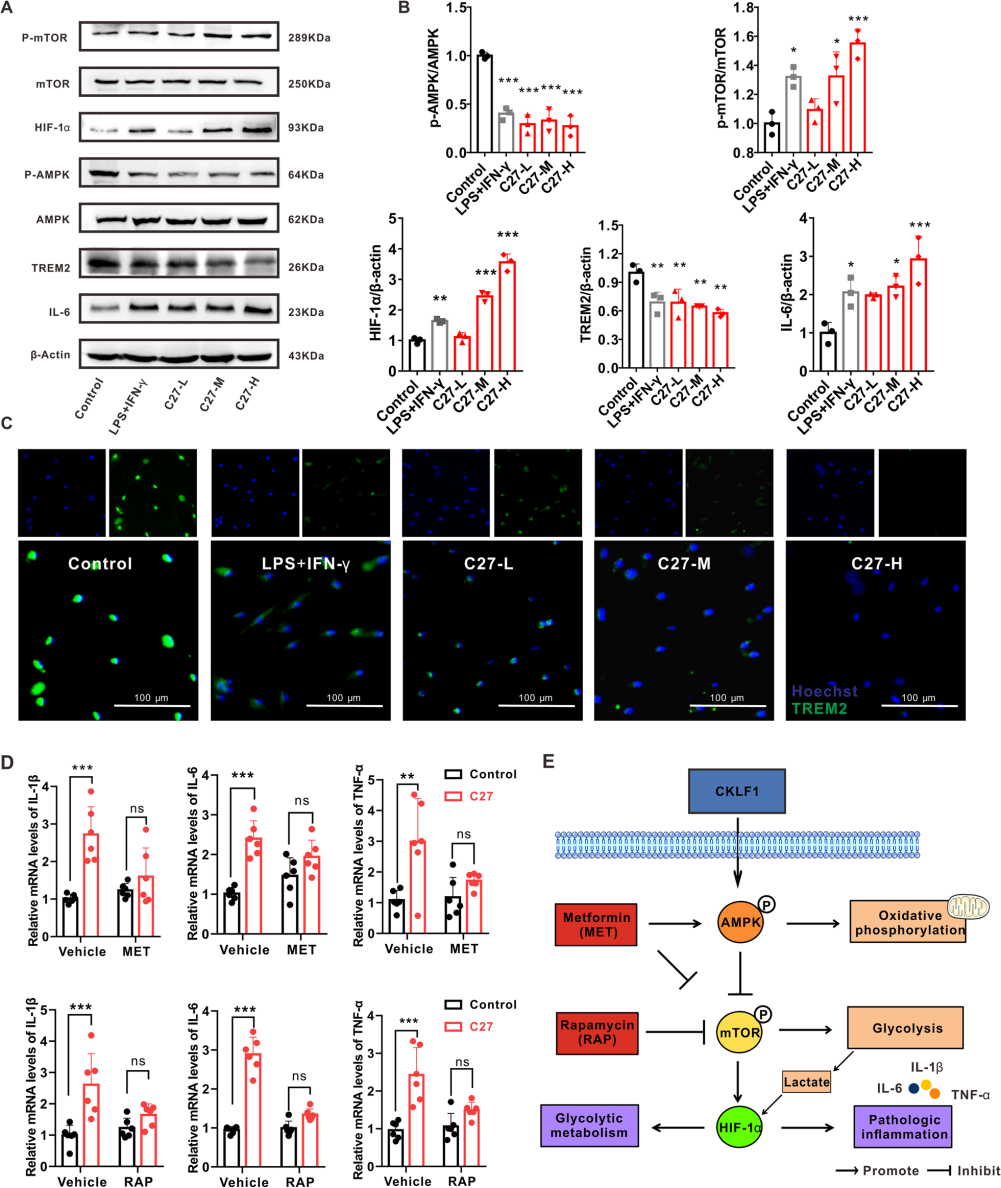
CKLF1-induced glycolysis and inflammation are dependent on activation of the AMPK–mTOR–HIF-1α pathway. **A** Representative image of the AMPK–mTOR–HIF-1α pathway. Microglia were treated with LPS + IFN-γ and various doses of C27 for 24 h, and immunoblot analysis of mTOR, p-mTOR, HIF-1α, AMPK, p-AMPK, TREM2, IL-6 and β-actin was performed (*n* = 3 per group). **B** Quantification of the gray values in **A** (*n* = 3 per group). p-AMPK/AMPK: *F* (4, 10) = 36.37; p-mTOR/mTOR: *F* (4, 10) = 12.76; HIF-1α/β-actin: F (4, 10) = 124.8; TREM2/β-actin: *F* (4, 10) = 9.472; IL-6/β-actin: *F* (4, 10) = 10.59.** C** Fluorescent signals of TREM2 induced by C27 (*n* = 3 per group). Scale bar = 100 μm. **D** Relative mRNA expression of IL-1β, IL-6 and TNF-α in microglia treated with C27 and rapamycin or metformin for 24 h (*n* = 6 per group). IL-1β: Vehicle: *F* (5, 5) = 41.99, MET: *F* (5, 5) = 17.03; IL-6: Vehicle: *F* (5, 5) = 8.296, MET: *F* (5, 5) = 1.189; TNF-α: Vehicle: *F* (5, 5) = 19.24, MET: *F* (5, 5) = 6.875. IL-1β: Vehicle: *F* (5, 5) = 9.089, RAP: *F* (5, 5) = 1.266; IL-6: Vehicle: *F* (5, 5) = 24.6, RAP: *F* (5, 5) = 1.601; TNF-α: Vehicle: *F* (5, 5) = 9.809, RAP: *F* (5, 5) = 2.741.** E** Schematic diagram of the effect of rapamycin or metformin on the AMPK–mTOR–HIF-1α pathway. The data are presented as the mean ± SD. *p < 0.05, **p < 0.01, ***p < 0.001 vs. control group

As the core of the cell's energy sensing mechanism, the mTOR pathway drives the glucose metabolism pathway. In this pathway, AMPK functions as a sensor for AMP and ADP, which indicate metabolic exhaustion, and inhibits mTOR phosphorylation. HIF-1α, which is the glycolysis master transcription factor, is induced by phosphorylated mTOR [35]. To investigate whether the mTOR pathway is involved in the metabolic reprogramming caused by CKLF1, we examined the AMPK–mTOR–HIF-1α pathways in PMG after CKLF1 treatment. As shown in Fig. 3A, CKLF1 exposure inhibited the phosphorylation of AMPK, which was followed by increased phosphorylation of mTOR. Importantly, CKLF1 dramatically increased HIF-1α levels relative to LPS/IFNγ (Fig. 3A, B). These results show that when microglia are exposed to CKLF1, the AMPK–mTOR–HIF-1α pathway is activated to promote glycolysis. Triggering receptor expressed on myeloid cells-2 (TREM2), which is a metabolic switch for microglia, was decreased when the mTOR pathway was activated (Fig. 3C). The loss of TREM2 has been shown to lead to metabolic deficits, including a reduction in mitochondrial respiratory capacity and an inability to perform glycolytic immunometabolic switching. It was also confirmed that CKLF1 exposure increased the level of IL-6 (Fig. 3A, B), suggesting that activation of the mTOR pathway was accompanied by an inflammatory response. Furthermore, blocking the mTOR pathway with rapamycin (an allosteric mTOR inhibitor) or metformin (an AMPK activator and mTOR inhibitor) reduced CKLF1-induced production of the pro-inflammatory cytokines IL-1β, IL-6 and TNF-α at the mRNA and protein levels (Fig. 3D). Our findings suggest that CKLF1-induced microglial inflammation is dependent on the AMPK–mTOR–HIF-1α pathway (Additional file 4: Fig. S3).

### Repeated exposure to CKLF1 results in metabolic abnormalities in microglia and immune tolerance

**Fig. 4:**
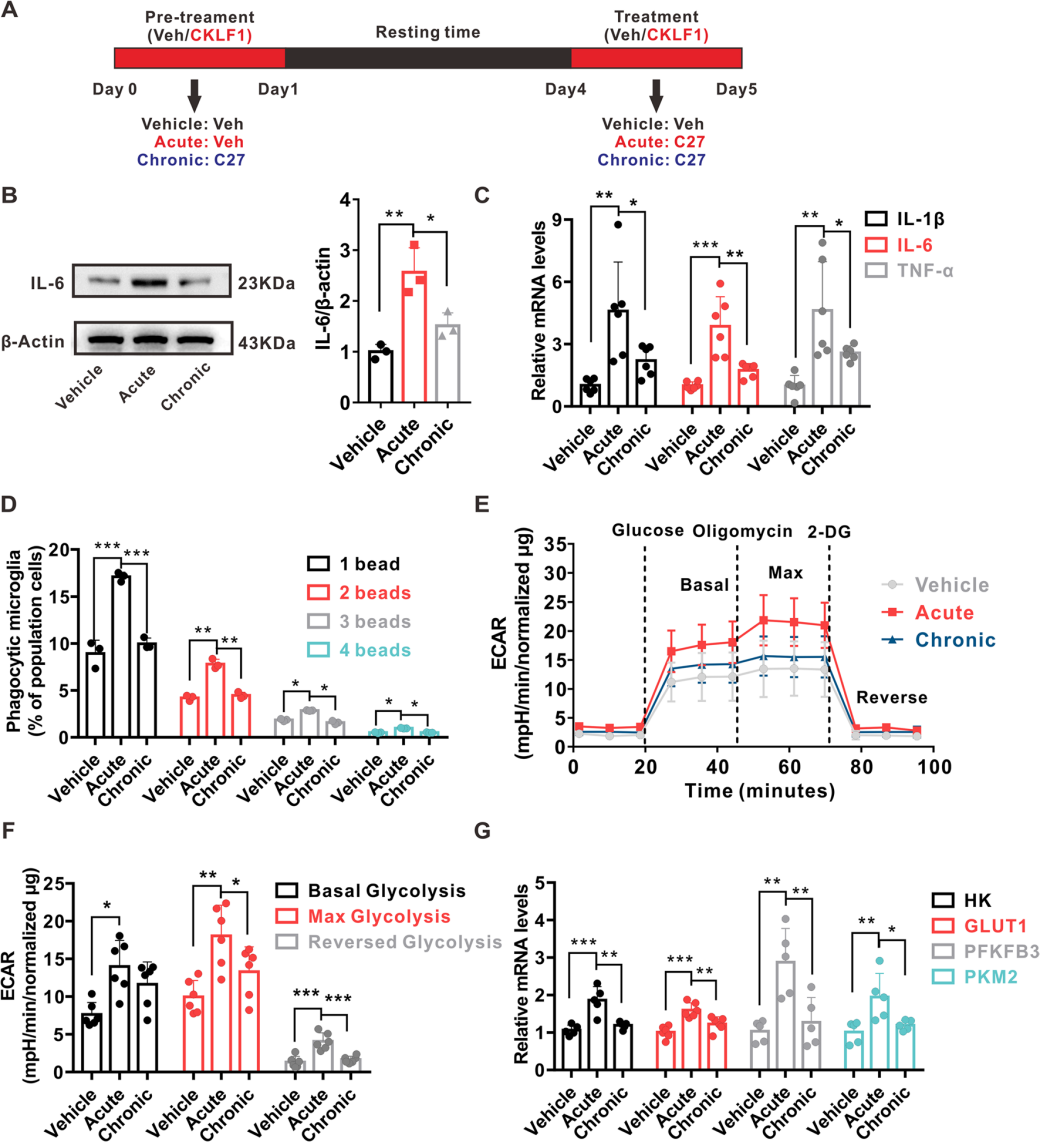
Repeated exposure to CKLF1-induced metabolic abnormalities and immune tolerance in microglia. **A** Timeline of repeated exposure of microglia to CKLF1. Microglia were stimulated by CKLF1 or vehicle for 24 h. After being washed, the cells were further cultured in medium without C27 for three days and then restimulated with CKLF1 or vehicle for 24 h, resulting in three experimental groups: Veh, acute, and chronic stimulation. **B** The level of IL-6 in microglia was determined by western blot (*n* = 3 per group). *F* (2, 6) = 17.97. **C** Relative mRNA levels of inflammatory cytokines in microglia (*n* = 6 per group). IL-1β: *F* (2, 15) = 9.64; IL-6: *F* (2, 15) = 18.18; TNF-α: *F* (2, 15) = 10.15. **D** Repeated exposure to C27 failed to induce phagocytosis in microglia. After microglia were induced with CKLF1, the amount of zymosan phagocytosed by cells in each group was detected by imaging and flow cytometry (*n* = 3 per group). 1 bead: *F* (2, 6) = 67.6; 2 beads: *F* (2, 6) = 78.46; 3 beads: *F* (2, 6) = 91.19; 4 beads: *F* (2, 6) = 108.5. **E**, **F** Repeated exposure to C27 led to metabolic abnormalities in microglia. The cell energy metabolism analyzer can detect real-time changes in extracellular acidification and quantitatively determine the basic glycolytic capacity, maximum glycolytic capacity and glycolytic reserve capacity to measure the ECAR (*n* = 6 per group). Basal glycolysis: *F* (2, 15) = 7.993; Max glycolysis: *F* (2, 15) = 9.294; Reversed glycolysis: *F* (2, 15) = 20.16. **G** Statistical analysis of the relative mRNA expression of glycolytic genes (*n* = 5–6 per group). HK: *F* (2, 12) = 17.34; GLUT1: *F* (2, 15) = 16.16; PFKFB3: *F* (2, 12) = 11.38; PKM2: *F* (2, 12) = 7.392. The data are presented as the mean ± SD. *p < 0.05, **p < 0.01, ***p < 0.001 vs. vehicle group or acute group

Given the prolonged expression of CKLF1 following stroke, we studied the influence of chronic CKLF1 on microglial metabolism. Within one day of exposure to danger signals, innate immune cells can be triggered or persistently adopt tolerance for the next 3 days, depending on the kind of stimulus. Tolerance can be induced by exposing cultured cells to stimuli for 24 h and then culturing the cells in the absence of further stimulation for 3 days. Thus, we stimulated PMG for 24 h with C27 or vehicle. PBS was used to wash away the drug, and after normal culture for 3 days, the cells were stimulated with CKLF1 or vehicle for 24 h for the second time, and the cells were divided into three experimental groups: Veh, Acute, and Chronic (Fig. 4A). The mRNA levels of IL-1β, IL-6 and TNF-α and the protein levels of the pro-inflammatory cytokine IL-6 were lower after chronic treatment with CKLF1 than after acute CKLF1 treatment and were comparable to those in unstimulated microglia (Fig. 4B, C). Furthermore, chronic CKLF1 treatment dramatically decreased the phagocytic function of microglia compared to that in the acute group (Fig. 4D, Additional file 4: Fig. S4C), indicating that CKLF1 induced innate immune tolerance in microglia.

To determine whether cellular metabolism plays a role in immune tolerance induced by chronic CKLF1 exposure, we examined the AMPK–mTOR pathway, in addition to glycolytic and OXPHOS metabolism in microglia. Using Seahorse, we determined that the ECAR induced by glucose was significantly improved, suggesting that basal glycolysis was enhanced in the acute group, but was downregulated in chronic treatment (“tolerance”). Moreover, oligomycin was used to inhibit the production of mitochondrial ATP to determine the maximum glycolysis, and it was found that the maximum glycolytic ability of microglia in the chronic group was significantly inhibited when compared to that in the acute group. The application of 2-DG to inhibit glycolysis showed that there was no significant difference among the vehicle, acute and chronic groups (Fig. 4E, F), suggesting that the variations in the ECAR in this study depended on the glycolysis. qPCR analysis showed that the increase in the expression of glycolysis-related genes by acute CKLF1 exposure was diminished in chronically treated cells (Fig. 4G), which provided additional evidence for their metabolic reprogramming.

The AMPK–mTOR–HIF-1α pathway, which induces acute inflammation in microglia, was downregulated in CKLF1-tolerant microglia (Fig. S3), ultimately leading to a decrease in the production of lactate and G6P (Fig. S4A and S4B). These results indicated that there were defects in glycolysis in tolerant cells. Mitochondrial dysfunction, which was observed in acutely activated microglia was observed in CKLF1-tolerant microglia (Fig. S4D and S4E). Additionally, sustained exposure increased necrotic microglia but not apoptotic microglia (Additional file 4: Fig. S4F, G). These observations suggest that broad defects in cellular metabolism including glycolysis and OXPHOS are present in CKLF1-tolerant microglia and lead to functional impairment of microglia.

### Genome-wide RNA sequencing of microglia isolated from the adult mouse brain identifies CKLF1-induced acute inflammation and tolerance

To evaluate whether chronic exposure to CKLF1 led to immune tolerance in microglia in vivo, C27 was administered directly to the M1 cortex by stereotaxic injection for four consecutive days. As shown in Additional file 5: Fig. S5, the first injection of C27 led to a pronounced increase in IL-1β, TNF-α and IL-10 (Additional file 5: Fig. S5). After the second injection, the level of IL-10 was decreased compared to that in the vehicle group, while IL-1β and TNF-α release occurred at similar levels. The increase in IL-1β and TNF-α was maintained until the third injection, and after the fourth injection of C27, the increased release of IL-1β and TNF-α was lost compared to that in the PBS group, indicating that the brain was immune tolerant.

**Fig. 5:**
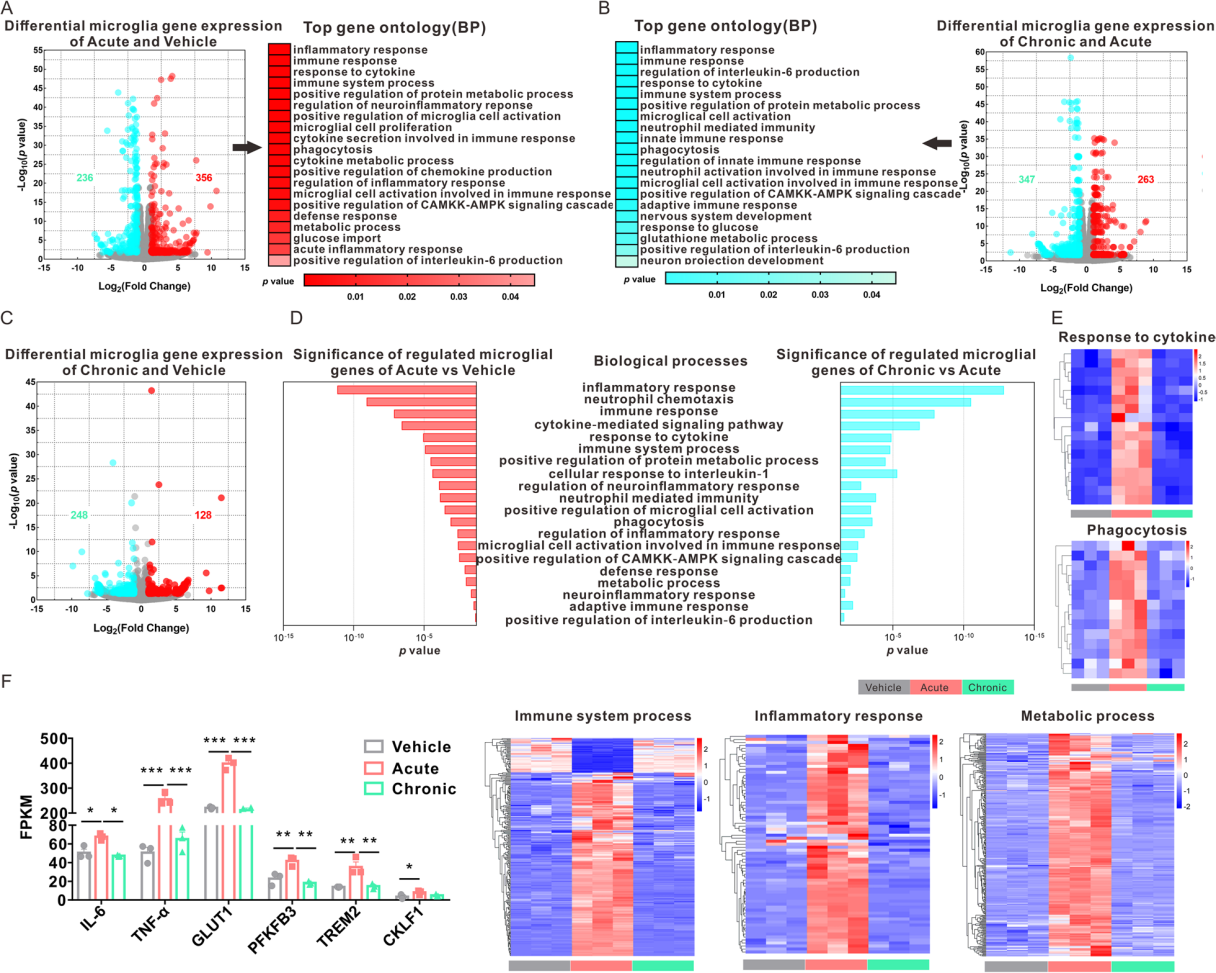
Gene expression in freshly isolated microglia from adult mice after acute or chronic stereotactic injection of C27. **A** Volcano plot showing changes in gene expression and upregulated genes in the microglia of C27 acutely stimulated mice that were significantly (*p* < 0.05) enriched for 20 BPs compared to PBS-treated mice. The importance of BPs is indicated by the intensity of red (*n* = 3 per group). **B** Volcano plots showing changes in gene expression and downregulated genes enriched in 20 significant (*p* < 0.05) BPs in the microglia of chronically C27-stimulated mice compared to acutely stimulated mice. The importance of the BPs is represented by the intensity of blue (*n* = 3 per group). **C** Volcano plot showing changes in gene expression the microglia of chronically C27-stimulated mice compared to PBS-treated mice (*n* = 3 per group). **D** Comparison of the significance of the top 20 BPs shared between the groups after acute and chronic treatment with C27 (*n* = 3 per group). **E** Heatmap depicting the transcriptional profile of selected BPs among the top 20 BPs (*n* = 3 per group). **F** Normalized expression of selected genes (*n* = 3 per group). IL-6: *F* (2, 6) = 18.14; TNF-α: *F* (2, 6) = 131.6; GLUT1: *F* (2, 6) = 117.1; PFKFB3: *F* (2, 6) = 18.82; TREM2: *F* (2, 6) = 13.66; CKLF1: *F* (2, 6) = 4.202. The data are presented as the mean ± SD. *p < 0.05, **p < 0.01, ***p < 0.001 vs. vehicle group or acute group

Moreover, we performed genome-wide RNA sequencing (RNA-seq) on freshly isolated microglia from mice 24 h after stereotactic injection of PBS or CKLF1 or with acute inflammation and tolerance induced for 4 consecutive days. Compared to PBS-treated animals, 356 upregulated genes and 236 downregulated genes were identified in acute CKLF1-treated mice (Fig. 5A). Among the upregulated genes, significantly (*p* < 0.05) enriched biological processes (BP) were identified and are shown as a heatmap. The term "inflammation response" includes genes involved in the immune response or the response to cytokines and was the most significantly enriched BP induced by acute CKLF1 injection. These findings suggest that acute exposure to CKLF1 in vivo leads to microglial inflammation and an enhanced immune response.

Subsequently, we analyzed changes in microglial gene expression between mice that were treated with chronic and acute CKLF1 injection. Compared with that in the acute stimulation group, we found 263 significantly upregulated genes and 347 significantly downregulated genes among the differentially expressed genes (Fig. 5B). Among the downregulated genes, inflammation response, immune response and regulation of IL-6 production were the most enriched BP induced by chronic CKLF1. In addition, the differentially expressed genes in the tolerance group and the vehicle group included 128 upregulated genes and 248 downregulated genes (Fig. 5C). Compared with the acute stimulation group, the overlap with the vehicle group was reduced. The differences between the first 20 genes in the acute and vehicle groups and the common differences in BP between the chronic and acute groups were compared. "Inflammatory response", "innate immune response", "cytokine response", "neutrophic chemotaxis" and “immune system function regulation” had significantly reduced correlations (Fig. 5D). We also observed different alterations in microglial genes in acute and tolerant animals (Fig. 5E). These factors enriched the BP genome-wide transcriptional spectrum (inflammatory response", "innate immune response", "response to cytokines", "phagocytosis", and "metabolic process" systemic processes) and associated selected genes (IL-6, TNF-α, GLUT1, PFKFB3, TREM2 CKLF1; Fig. 5F) and showed that microglia in the brains of tolerant mice were not activated and less reactive than microglia from mice with CKLF1-stimulated acute inflammation.

### Immune-tolerant microglia trained by CKLF1 lose the ability to phagocytose neutrophils

**Fig. 6:**
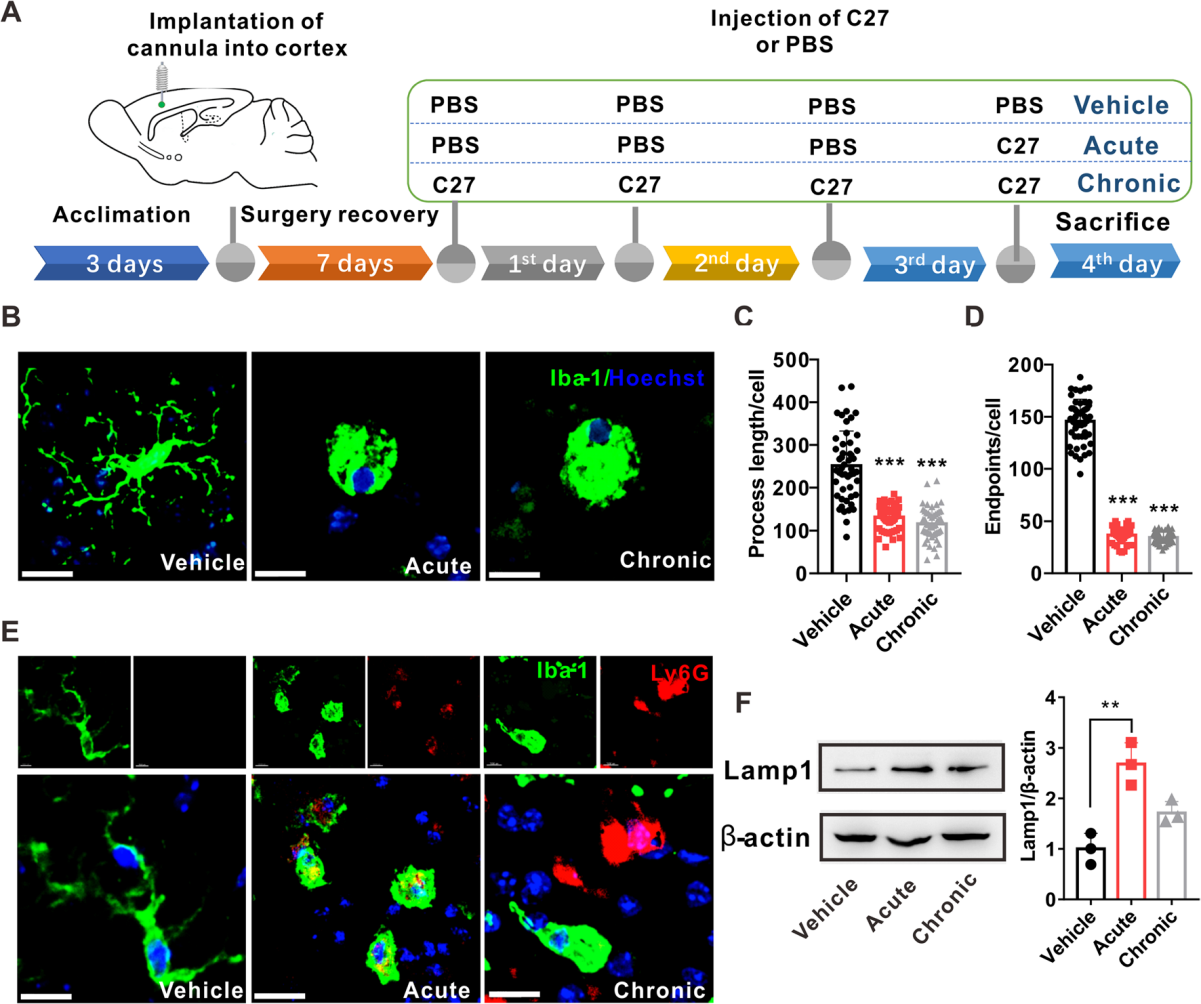
Chronic administration of C27 to the M1 cortex produced immune tolerance in vivo. **A** C27 was delivered into the brain at a dose of 10 μg through stereotactic injection once or four times, and the tissues were collected 24 h after the last injection. **B** The structure of microglia was revealed by Iba-1 staining, which was further analyzed by skeleton analysis in ImageJ. Scale bar = 10 μm. **C** Analysis of process lengths induced by C27 (*n* = 50 per group). *F* (2, 147) = 93.57. **D** Analysis of the number of endpoints per microglia induced by C27 (*n* = 50 per group). *F* (2, 147) = 1149. **E** Microglia could not phagocytose neutrophils after chronic administration of C27. Microglia and neutrophils were stained green and red, respectively, and the merged images showed neutrophils engulfed by microglia (*n* = 3 per group). Scale bar = 15 μm. **F** Lamp1 was decreased by chronic administration of C27 (*n* = 3 per group). *F* (2, 6) = 19.74. The data are presented as the mean ± SD. **p < 0.01, ***p < 0.001 vs. vehicle group

To examine how microglia respond to CKLF1 in vivo, we injected PBS or C27 into the cerebral cortex. For acute stimulation, the drug was given only once, and for chronic stimulation, it was administered for 4 consecutive days (Fig. 6A). Microglia underwent marked morphological changes after acute stimulation with C27 (Fig. 6B), and resting microglia were highly branched and uniformly distributed throughout the brain parenchyma. The structure of microglia was revealed by Iba-1 staining, which was further analyzed by skeleton analysis in ImageJ (Fig. 6C, D). The results demonstrated that acute CKLF1 administration caused substantial changes in microglial morphology, including reduced process lengths and endpoints while chronic CKLF1 treatment also resulted in larger cell bodies, as characterized by decreased endnotes and process length, and there were no obvious differences after acute and chronic stimulation of CKLF1.

CKLF1 has been shown to induce neutrophil chemotaxis. We previously showed that CKLF1 can exacerbate neutrophil infiltration after stroke, and infiltrated neutrophils can be phagocytosed and cleared by microglia. In this study, we used green fluorescence to label microglia and red fluorescence to label Ly6G, which is a marker protein of neutrophils. The results showed that acute CKLF1 administration significantly increased the co-localization of neutrophils and microglia, indicating that microglia could phagocytose neutrophils. The number of neutrophils in the chronic CKLF1 group was significantly higher than that in the acute CKLF1 group, and the level of yellow fluorescence was significantly reduced, indicating that the ability of microglia to phagocytose neutrophils was reduced (Fig. 6E). These results demonstrate that long-term administration of CKLF1 induced microglia into a state of immune tolerance. Furthermore, the levels of Lamp1, which is a biomarker of lysosomes, were also decreased in the chronic group compared to the acute group (Fig. 6F), suggesting that the degradation capacity of microglia was also diminished.

### The loss of CKLF1 restores microglial phagocytosis and improves the long-term outcomes of stroke

**Fig. 7:**
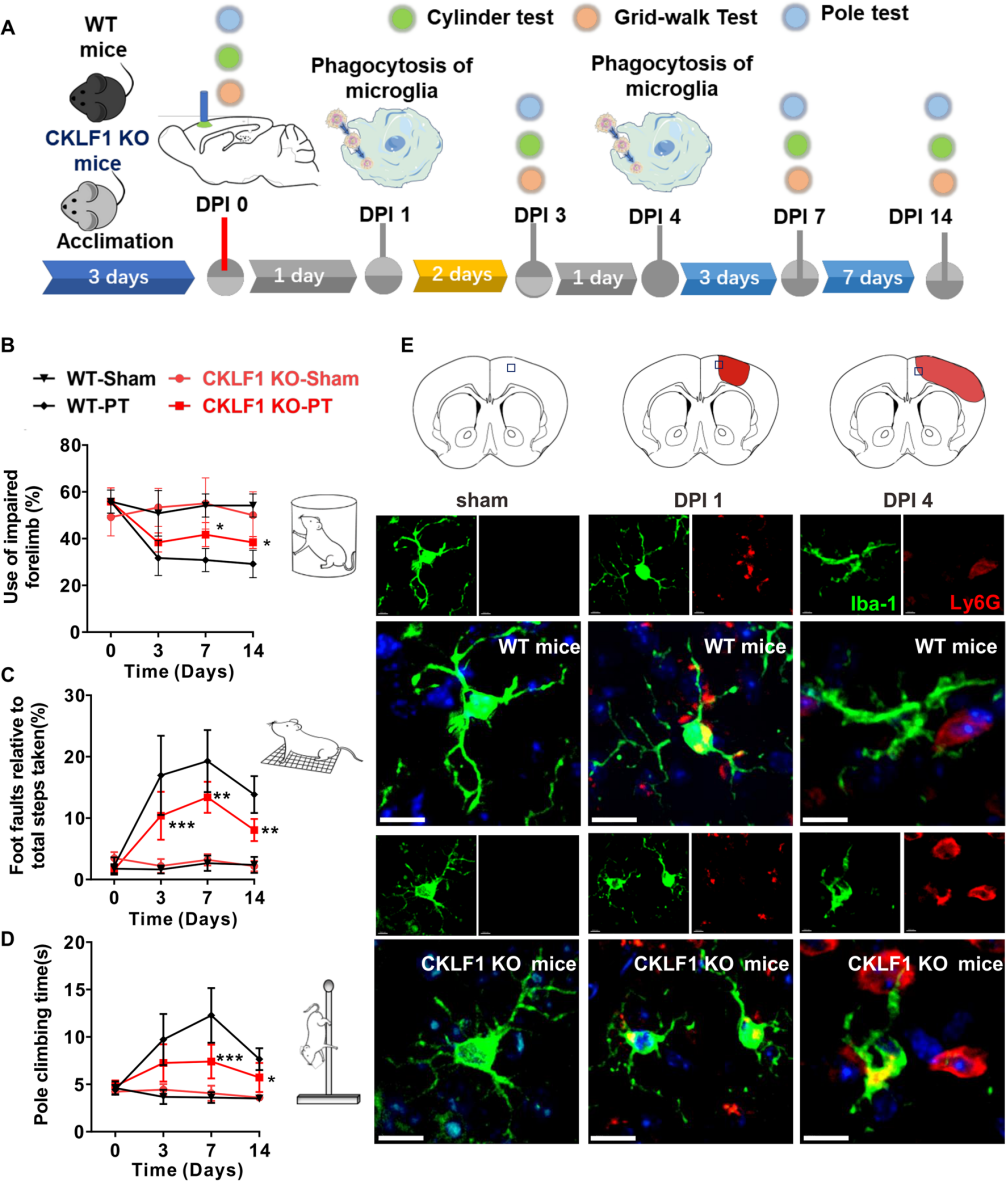
KO of CKLF1 improves behavioral performance and restores microglial phagocytosis after stroke. **A** Schematic diagram showing that the loss of CKLF1 restores microglial phagocytosis and improves the long-term outcomes of stroke established by photothrombosis. **B**–**D** Functional recovery in mice was assessed by the cylinder test (**B**), grid walking task (**C)** and pole climbing test (**D)** at baseline and 3, 7 and 14 days after stroke. (*n* = 6 per group), the data are presented as the mean ± SD, * *p* < 0.05, ** *p* < 0.01, *** *p* < 0.001 vs. WT-PT group at corresponding time point. Interaction F (DFn, DFd): cylinder test: *F* (9, 80) = 5.775; grid walking task: *F* (9, 80) = 12.57; pole climbing test: *F* (9, 78) = 9.305. **E** CKLF1 KO maintains neutrophil phagocytosis. Microglia were stained with an antibody against Iba-1 (green), nuclei were stained with Hoechst (blue), and neutrophils were stained with Ly6G (red). Typical images of morphology of phagocytosis of neutrophils by microglia in WT and CKLF1 KO mice (*n* = 3 per group). Scale bar = 15 μm

To confirm the biological role of CKLF1-induced immunological tolerance in microglia following stroke, we used gene knockdown and antibody neutralization to inhibit the biological activity of CKLF1 in mice with photothrombotic stroke. In CKLF1-KO mice, we observed dramatic improvements in motor dysfunction, as characterized by considerably reduced mistake rates in the cylinder test in compared to wild-type (WT) mice from 7 to 14 DPI (Fig. 7B). The grid-walk test showed a dramatic reduction in the number of mistakes in CKLF1-KO mice compared with WT mice from 3 to 14 DPI (Fig. 7C). Furthermore, the improvements in motor function due to the loss of CKLF1 was also observed in pole test, which was characterized by a decrease in the pole climbing time from 7 to 14 DPI (Fig. 7D). All of results indicated that loss of CKLF1 improved the outcome of photothrombotic stroke. In addition, the loss of CKLF1 greatly increased the phagocytosis of infiltrating neutrophils by microglia, as evidenced by an increase in the co-localization of microglia and neutrophils in the ischemic marginal zone (Fig. 7E), suggesting that immune tolerance after stroke could be prevented by the loss of CKLF1.

### Short-term neutralization of CKLF1 produced long-term improvement in behavioral performance in photothrombotic stroke

**Fig. 8:**
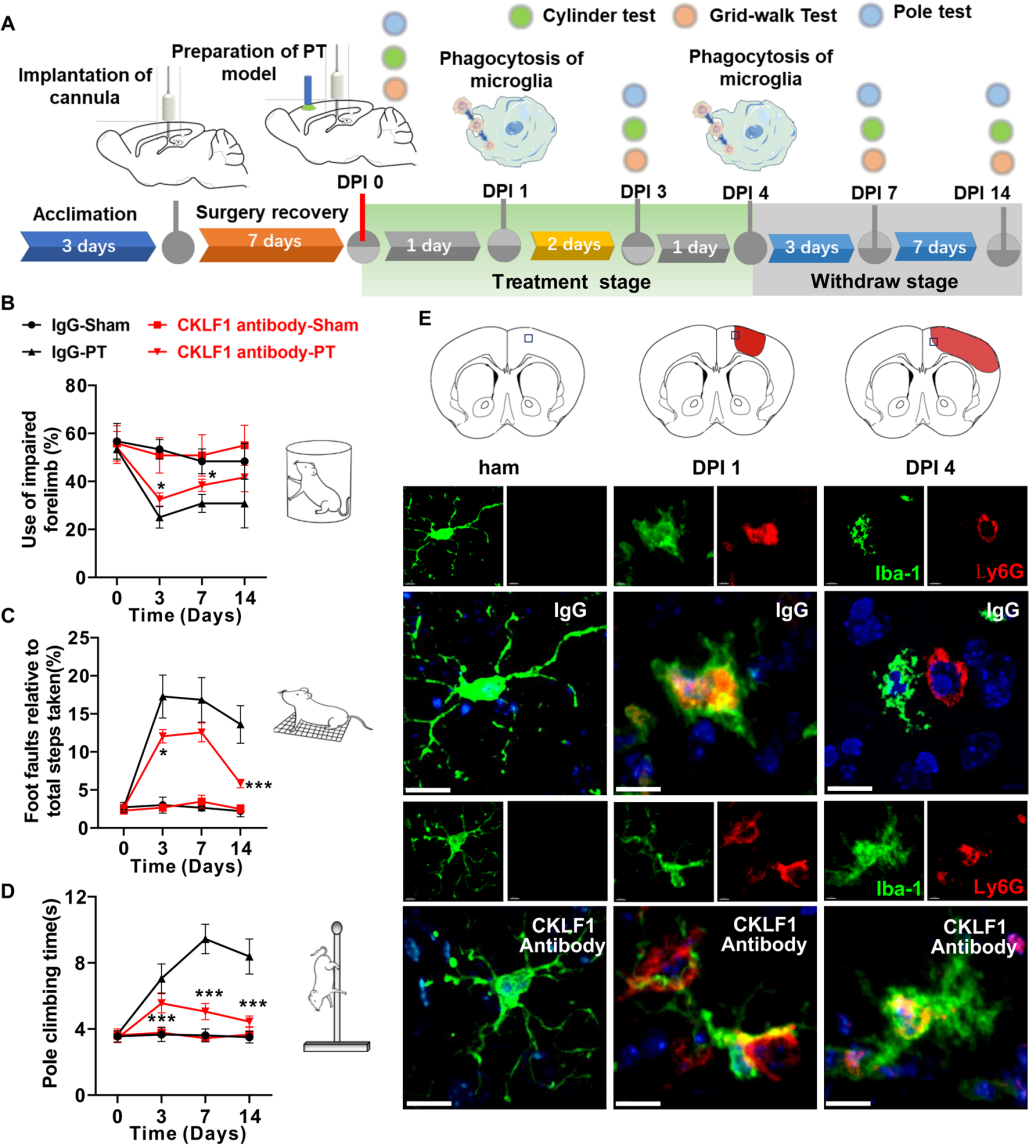
Short-term neutralization of CKLF1 produced long-term improvements in behavioral performance and restored microglial phagocytosis after stroke. **A** Schematic diagram showing that the neutralization of CKLF1 restored microglial phagocytosis and improved the long-term outcomes of stroke established by photothrombosis. **B**–**D** Functional recovery was assessed by the cylinder test (**B**), grid walking task (**C)** and pole climbing test (**D)** at baseline and 3, 7 and 14 days after stroke (*n* = 6 per group), *p < 0.05, ** *p* < 0.01, *** p < 0.001 *vs.* IgG-treated group at the corresponding time point. Interaction F (DFn, DFd): cylinder test: *F* (9, 60) = 3.947; grid walking task: *F* (9, 60) = 40.09; pole climbing test: *F* (9, 60) = 28.37. **E** Neutralization of CKLF1 maintains neutrophil phagocytosis at 1 and 4 DPI. Microglia were stained with an antibody against Iba-1 (green), nuclei were stained with Hoechst (blue), and neutrophils were stained with Ly6G (red). Typical images of morphology of phagocytosis of neutrophils by microglia in IgG and CKLF1 antibody-treated mice. Scale bar: 15 μm

To exclude the likelihood that CKLF1 deficiency may result in aberrant brain function, we blocked CKLF1 activity by injecting a CKLF1-neutralizing antibody into the lateral ventricle of the brain. The expression of CKLF1 in ischemic stroke began 8 h after stroke and peaked 2 to 3 days after stroke, accordingly, the treatment window for the CKLF1-neutralizing antibody was defined as four days following stroke, which included the peak period of CKLF1 production and neutrophil infiltration. Compared to those in the IgG control group, mice in the CKLF1-neutralizing antibody group exhibited considerably better behavior, and the phagocytic activity of microglia was significantly higher than that in the IgG control group. In the cylinder test, neutralizing to CKLF1 significantly improved the percent of impaired forelimb use compared to that in the IgG group at 3 and 7 DPI (Fig. 8B). The grid test showed that there was a marked reduction in foot faults in the CKLF1 antibody-treated animals at 3 and 14 DPI (Fig. 8C). The pole test revealed that blocking CKLF1 activity significantly reduced the pole climbing time (Fig. 8D). More importantly, this improvement persisted for 2 weeks, indicating that short-term blockade of CKLF1 could produce a long-lasting effect after stroke. In addition, neutralizing CKLF1 greatly increased the phagocytosis of infiltrating neutrophils by microglia, as evidenced by an increase in the co-localization of microglia and neutrophils in the ischemic marginal zone (Fig. 8E), which provides evidence for the principal role of CKLF1 in the immune tolerance of microglia following stroke.

## Discussion

Regulating the inflammatory response is one of the important pathways for the treatment of neuronal damage caused by stroke. This study showed that acute exposure of CKLF1 to microglia promotes its phagocytosis by reprogramming their cellular metabolism. On the other hand, chronic treatment to CKLF1 turned microglia into immune-tolerant state, resulting in the loss of phagocytic function to the infiltrated neutrophils and aggravation of inflammatory injury. Therefore, the dual response induced by CKLF1 in microglia may represent an important processing in the inflammatory response post-stroke.

Microglia are the first line of defense for immune defense in the central nervous system. In our previous studies, we showed that CKLF1 induced the release pro-inflammatory cytokines from microglia, aggravated the inflammatory response 24-h after stroke [25]. However, the effect of CKLF1 on the progression of inflammatory response in the acute phase after stroke has not yet been elucidated. According to the expression kinetics of CKLF1 after stroke, CKLF1 shows an inverted U-shaped expression curve in the ischemic brain regions, and it peaks on the third day after stroke [36]. Previous studies have shown that immune cells can develop immune tolerance within a continuous activation period of 3 to 5 days [37]. Therefore, we first observed the effect of CKLF1 on microglia activation at different times. C27, the C-terminal active peptide of CKLF1, has been proved to be able to interact with receptors such as CCR4 to induce the immune response [27]. Our results show that acute incubation of C27 activates microglia, manifested as increased phagocytosis and significantly increased cytokine production. IL-6 is an important cytokine for post-stroke increase. Studies have shown that IL-6 is not only an independent risk factor for post-stroke neuropathological damage, but is closely related to stroke recurrence [38–40]. Our study showed that acute exposure to C27 caused a time-dependent increase in microglia IL-6 expression. In the phagocytic test, we found that C27 not only increased the proportion of phagocytosed microglia, but also significantly increased their phagocytic ability. These two aspects indicated that CKLF1 could lead to microglial activation.

An increasing number of studies have shown that the interaction between metabolism and immune function has an important effect on cell function [27]. Metabolic reprogramming refers to the reuse of enzymes and metabolites to control various physiological and biochemical functions of cells through cell signal transduction pathways. It has confirmed that metabolic reprogramming plays an important role in inflammation and the polarization of immune cells [41].

In this study, we found that CKLF1 significantly enhanced the glycolysis ability of microglia and inhibited the oxidative phosphorylation pathway. In the glycolytic performance test, we showed that CKLF1 significantly increased the basic glycolytic and maximum glycolytic abilities of microglia. In the measurement of oxidative phosphorylation, CKLF1 inhibited the basic oxidative phosphorylation and maximum oxidative phosphorylation of microglia, indicating that the energy metabolism pathway of microglia was recombined. Different from glycolysis, oxidative phosphorylation can also use glutamine and short-chain fatty acids as energy substrates to enter the tricarboxylic acid cycle except for glucose [42]. Although the oxidative phosphorylation caused by CKLF1 was significantly reduced, whether this reduction was result from the preference changes for its energy substrate needs to be further studied.

Increased glucose uptake is a prerequisite for enhanced glycolysis [43]. Up to now, more than 10 members in the glucose transporter family have been found [44]. The glucose transporters that are abundantly expressed in microglia are Glut1, Glut3, Glut5, etc. BV-2 and mouse primary microglia were selected cell types in this study, in which Glut5 was not expressed. The increased expression of Glut1 is necessary for microglia activation, and Glut3 is not involved in this process, suggesting that Glut1 may be a potential transporter that CKLF1 induced microglia activation [45]. In this study, we found that C27 significantly increased the expression of Glut1, which promoted glucose uptake and provided more substrates for the promotion of glycolysis.

Excessive glucose in the cytoplasm increased mitochondrial pressure [46]. In this study, it was found that CKLF1 may promote mitochondrial fission by their morphological analysis. Mitochondrial fission is a process of mitogenesis and also be a pathway for mitochondria to separate from the damaged residuals. In order to distinguish two biological processing, we detected the changes of pyruvate dehydrogenase, which was related to mitochondrial respiratory chain [47], and found that high concentration of CKLF1 decreased pyruvate dehydrogenase activity, suggesting that mitochondrial fission might not be able to promote mitochondrial regeneration. The reduced OCR values also indicated that mitochondrial fission did not associate with the increased function of healthy mitochondria. The above result indicated that CKLF1-induced mitochondrial fission was not related to mitochondrial regeneration, but might be a processing that clears away damaged mitochondrial residuals.

To observe the biological role of elevated glycolysis in CKLF1-induced microglial activation, we first used the glycolysis inhibitor 2-DG to observe and found that 2-DG significantly blocked C27-induced microglia activation. However, 2-DG is controversial as a blocker of glycolysis, manifested by it not only on glycolysis, but also on oxidative phosphorylation, as demonstrated by its ability to promote oxidative phosphorylation at concentrations less than 1.25 mM and to inhibit oxidative phosphorylation at concentrations up to 10 mM [34]. In this study, we chose 4 mM as the working concentration of 2-DG in vitro, a concentration that was effective in inhibiting glycolysis without significant effects on oxidative phosphorylation. On this basis, we observed that 2-DG blocked C27-increased inflammatory factors (IL-6, IL-1β and TNF-α) and inhibited C27-enhanced microglial phagocytosis, indicating that glycolysis is necessary for C27-induced microglial activation. However, it has been shown that 2-DG can block the expression of M2-type markers and affect the polarization state of microglia [34]. In order to avoid this confounding effect of 2-DG, we chose another glycolysis inhibitor, galactose, which must be metabolized by the Leloir pathway before entering glycolysis, leading to profound reduction in glycolytic flux and functionally effective inhibitory slow down. The results showed that application of galactose also blocked C27-induced microglia activation, proving from another aspect that glycolysis is necessary for C27-activated microglia.

Furthermore, we observed the microglial performance who was chronic exposure to CKLF1. It has been proved that both Aβ and synuclein could lead to microglia into immune tolerance after 3 days of first stimulation [48]. The peak of CKLF1 expression after stroke is 2 to 3 days, hence, we chose to observe the effect of repeated administration of CKLF1 on microglia function. Our results showed that repeated administration of CKLF1 did not induce microglial activation when compared with single exposure of CKLF1,characterized by the reduced cytokine production and phagocytosis, suggesting that microglia enter a state of immune tolerance. Energy metabolism analysis showed that repeated administration of CKLF1 not only failed to enhance glycolysis, but also reduced basic and maximum glycolysis abilities significantly when compared with acute CKLF1 stimulation. The detection of OCR showed that repeated administration of CKLF1 induced further damage to OCR, indicating that mitochondrial function was also impaired, which also verified that mitochondrial fission was not the regeneration of healthy mitochondria, but the degradation pathway of mitochondrial autophagy in damaged mitochondria.

To investigate the effect of CKLF1 on the immune states of microglia in vivo, we performed genome-wide RNA sequencing using acute microglia isolation and found that acute administration of CKLF1 significantly activate microglia, and the upregulated genes were enriched in such biological processes as "inflammatory response" and "immune response", indicating that microglia were immune-activated. Compared with acute administration of CKLF1, microglia isolated from the chronic CKLF1-treated mice enriched the downregulated genes in such biological processes as "inflammatory response", "immune response", and "regulation of IL-6 expression", indicating that long-term exposure to CKLF1 could lead to immune tolerance of microglia. Previous studies have shown that microglia deficiency can lead to the accumulation of neutrophils after stroke, which in turn aggravates the neuroinflammatory response [49]. Our study found that microglial immune tolerance may be another pathway for CKLF1 to participate inflammation in the post-stroke stage.

To observe the effect of microglia immune state caused by CKLF1 on stroke outcome, gene knockout and antibody neutralization were, respectively, observed in this study, and the results showed that either inhibition of CKLF1 expression or blocking its activity could significantly improve motor dysfunction in stroke mice and enhance the phagocytic function of microglia on neutrophils, indicating that CKLF1 is a potential target for the treatment of stroke. It should be noted that blocking the activity of CKLF1 in a short-term produced benefit the locomotor function of mice after stroke for a long time, which may be related to the expression manner that CKLF1 belongs to inducible genes, absent in the adult healthy brain. It is known that the expression of CKLF1 is time-dependent after stroke, and it is almost undetectable in 7 days after stroke [23]. Therefore, taking CKLF1 as a therapeutic target for stroke may has the advantage of short treatment stage.

It is also important that CKLF1-induced glycolysis was dependent on the regulation of AMPK/mTOR signaling pathway, and both mTOR and AMPK were once considered as potential therapeutic targets for the treatment of stroke. Metformin, an AMPK agonist, is widely used in the treatment of type 2 diabetes and has been proven to be useful for preventing the occurrence of stroke. However, there is no significant therapeutic effect in patients with type 2 diabetic stroke [50, 51]. mTOR has demonstrated good clinical efficacy as a therapeutic target for CNS diseases in neurodevelopmental disorders such as tuberous sclerosis. However, there are still no definitive studies in the treatment of cerebral hemorrhage [52]. Compared with them, CKLF1, as an ischemia-induced expression protein, participates in the regulation of microglia immune state, which has the potential advantages of short treatment stage and long efficacy time.

## Conclusions

In summary, our findings suggest that metabolic reprogramming is the basis of CKLF1-induced microglial inflammation, which was dependent on the AMPK/mTOR pathway. In addition, CKLF1-tolerant microglia show metabolic dysfunction and phagocytic dysfunction. Knocking out or blocking the activity of CKLF1 restores microglial activation and increases neutrophil phagocytosis after photothrombotic stroke. Overall, CKLF1 plays a crucial role in the relationship between microglial metabolic status and immune function in stroke, which prepares a potential therapeutic strategy for ischemic stroke.

## Supplementary Information

**Additional file 1: Figure S1**. CKLF1 induces activation of microglia.** A** The purity of PMG. Scale bar = 50 μm.** B** The level of IL-6 mRNA stimulated by C27. F = 17.06.** C** Exposure of C27 increased the phagocytosis of microglia. Microglia were treated with LPS + IFN-γ and each dose of C27 for 24 h, and the amount of zymosan phagocytosed by cells in each group was detected by imaging flow cytometry. F = 11.72.** D** Relative mRNA level of M1-type polarization markers in microglia. CD16: F = 4.854; CD32: F = 7.735; iNOS: F = 3.205.** E** Relative mRNA expression of M2-type polarization markers in microglia. Arg1: F = 1.416; TGF-β: F = 2.027; CCL-22: F = 3.881.** F** Fluorescence imaging of TUNEL staining and quantification of positive cells. F = 3.563. Scale bar = 50 μm. G The amount of lactate dehydrogenase released from the microglia. F = 7.709. Data are presented as mean ± SD. *p < 0.05, **p < 0.01, ***p < 0.001 vs. control group.

**Additional file 2: Figure S2**. CKLF1 impairs mitochondrial function and morphology.** A**,** B** The levels of lactate and pyruvate kinase in microglia after C27 treatment. Lactate: F = 13.83; pyruvate kinase: F = 10.34.** C** The levels of glucose-6-phosphate in microglia after C27 treatment. F = 4.845.** D** Relative mRNA level of responsible for pyruvate transport MPC1 and MPC2. MPC1: F = 7.878; MPC2: F = 3.411.** E** The levels of pyruvate dehydrogenase activity in microglia after C27 treatment. F = 3.08.** F** Representative image of mitochondrial morphology in living cells. Scale bar = 20 μm.** G** Analysis of mitochondrial morphology describes by the shape factor and aspect ratio. Form factor: F = 3.856; Aspect ratio: F = 3.041. Data are presented as mean ± SD. *p < 0.05, **p < 0.01, ***p < 0.001 vs. control group.

**Additional file 3: Figure S3**. Repeated exposure of C27 failed to activate the AMPK–mTOR–HIF-1α signaling pathway.** A** Representative image of mTOR, p-mTOR, HIF-1α, AMPK, p-AMPK and β-actin in microglia.** B** Quantification of gray values of western blot results. p-AMPK/AMPK: F = 15.1; p-mTOR/mTOR: F = 28.89; HIF-1α/β-actin: F = 20.88. Data are presented as mean ± SD. **p < 0.01, ***p < 0.001 vs. vehicle group.

**Additional file 4: Figure S4**. Repeated exposure of CKLF1-induced metabolic abnormalities and immune tolerance in microglia.** A** The level of lactate in microglia. F = 11.89.** B** The level of glucose-6-phosphate in microglia. F = 9.057.** C** Chronic stimulation of C27 led to failure of phagocytosis in microglia. The amount of zymosan phagocytosed by microglia was detected by imaging flow cytometry. F = 25.41.** D** Real-time changes in the oxygen consumption rate of cells.** E** Quantitative statistics of the basal respiration, proton leak, and maximal respiration. Basal respiration: F = 1.368; proton leak: F = 3.365; maximal respiration: F = 8.349.** F** Quantification of TUNEL staining induced by C27. Scale bar = 50 μm. F = 3.756.** G** The amount of LDH released from the microglia. F = 10.4. Data are presented as mean ± SD. *p < 0.05, **p < 0.01, ***p < 0.001 vs. vehicle or acute group.

**Additional file 5: Figure S5**. Repeated injection of C27 into M1 area of cortex reshaped the inflammation cytokines level. C27 was delivered into M1 cortex at the dose of 10 μg through stereotactic injection for different times, and the tissues was collected after 24 h of last injection. The levels of IL-1β, TNF-α and IL-10 were determined by ELISA. IL-1β: F = 89.68; TNF-α: F = 156.1; IL-10: F = 21.91. Data are presented as mean ± SD. *p < 0.05, **p < 0.01, ***p < 0.001 vs. PBS group.

## Data Availability

The datasets used and/or analyzed during the current study are available from the corresponding author on reasonable request.
