# Supplementary figures and images for: A breakdown of metabolic reprogramming in microglia induced by CKLF1 exacerbates immune tolerance in ischemic stroke
Source: J Neuroinflammation. 2023 Apr 25;20:97. doi: 10.1186/s12974-023-02779-w (PMC10127063; doi:10.1186/s12974-023-02779-w)

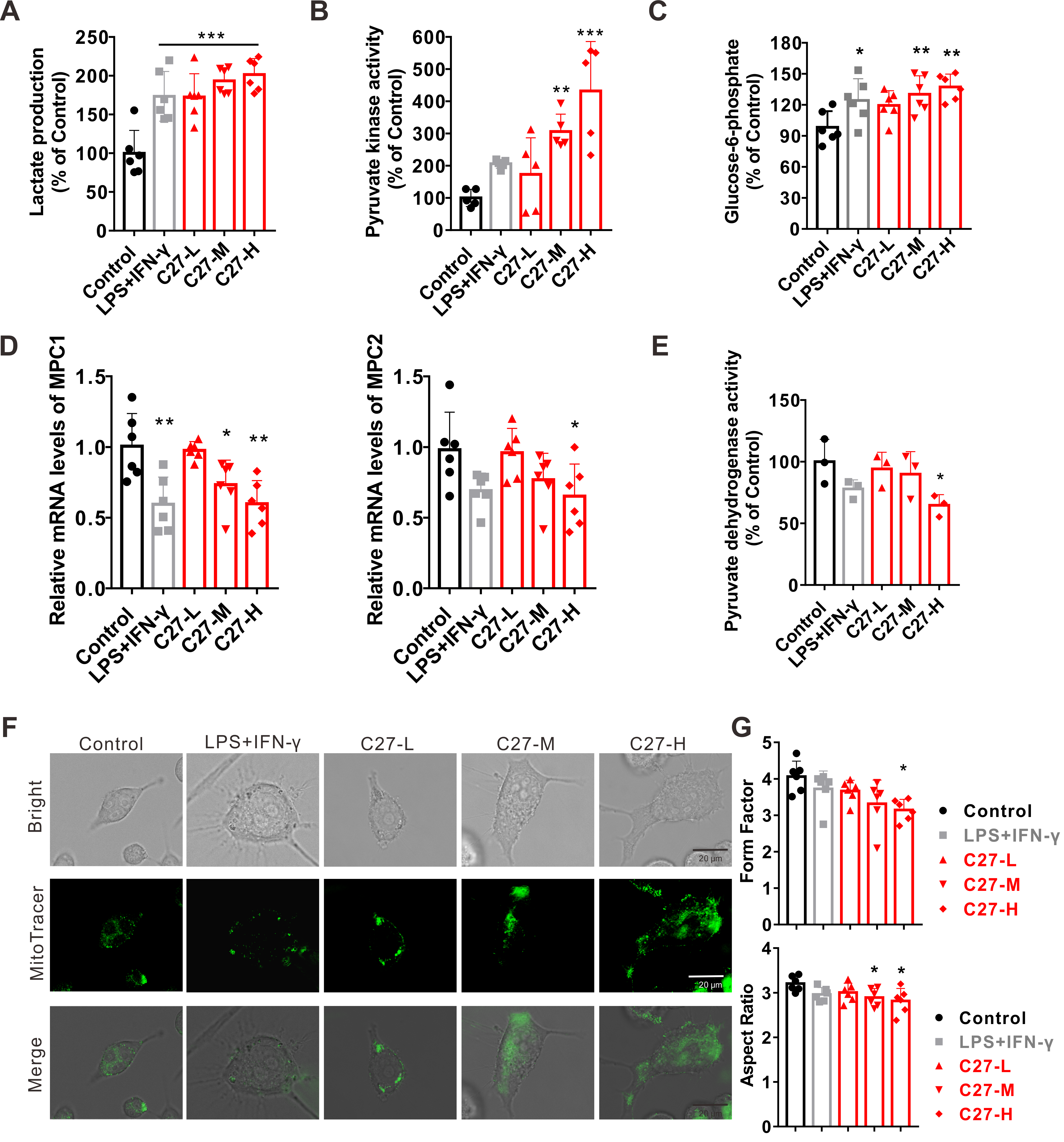

Supplement: Supplementary file 2 — Additional file 2: Figure S2. CKLF1 impairs mitochondrial function and morphology. A, B The levels of lactate and pyruvate kinase in microglia after C27 treatment. Lactate: F = 13.83; pyruvate kinase: F = 10.34. C The levels of glucose-6-phosphate in microglia after C27 treatment. F = 4.845. D Relative mRNA level of responsible for pyruvate transport MPC1 and MPC2. MPC1: F = 7.878; MPC2: F = 3.411. E The levels of pyruvate dehydrogenase activity in microglia after C27 treatment. F = 3.08. F Representative image of mitochondrial morphology in living cells. Scale bar = 20 μm. G Analysis of mitochondrial morphology describes by the shape factor and aspect ratio. Form factor: F = 3.856; Aspect ratio: F = 3.041. Data are presented as mean ± SD. *p < 0.05, **p < 0.01, ***p < 0.001 vs. control group. [file 12974_2023_2779_MOESM2_ESM.tif]

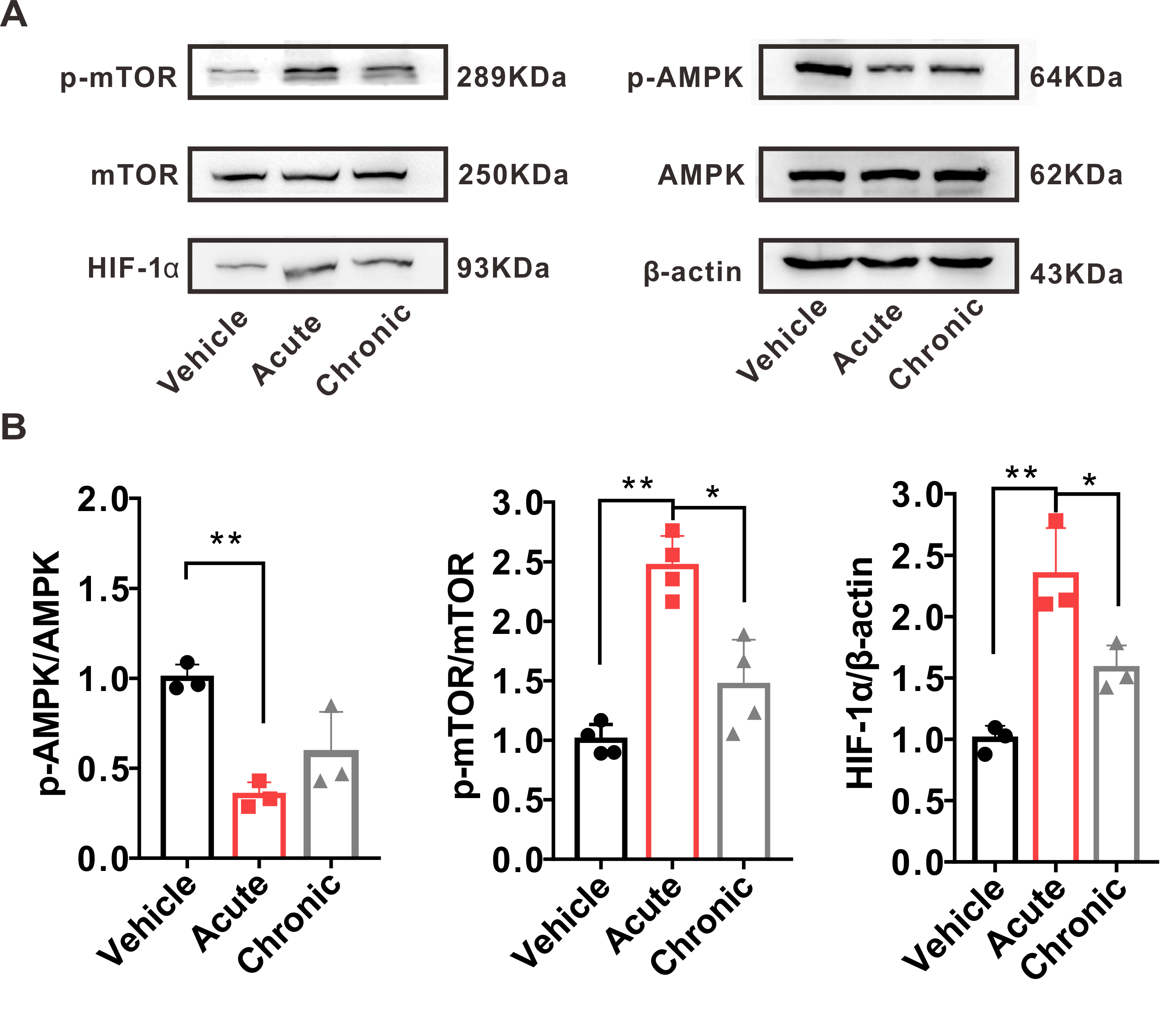

Supplement: Supplementary file 3 — Additional file 3: Figure S3. Repeated exposure of C27 failed to activate the AMPK–mTOR–HIF-1α signaling pathway. A Representative image of mTOR, p-mTOR, HIF-1α, AMPK, p-AMPK and β-actin in microglia. B Quantification of gray values of western blot results. p-AMPK/AMPK: F = 15.1; p-mTOR/mTOR: F = 28.89; HIF-1α/β-actin: F = 20.88. Data are presented as mean ± SD. **p < 0.01, ***p < 0.001 vs. vehicle group. [file 12974_2023_2779_MOESM3_ESM.tif]

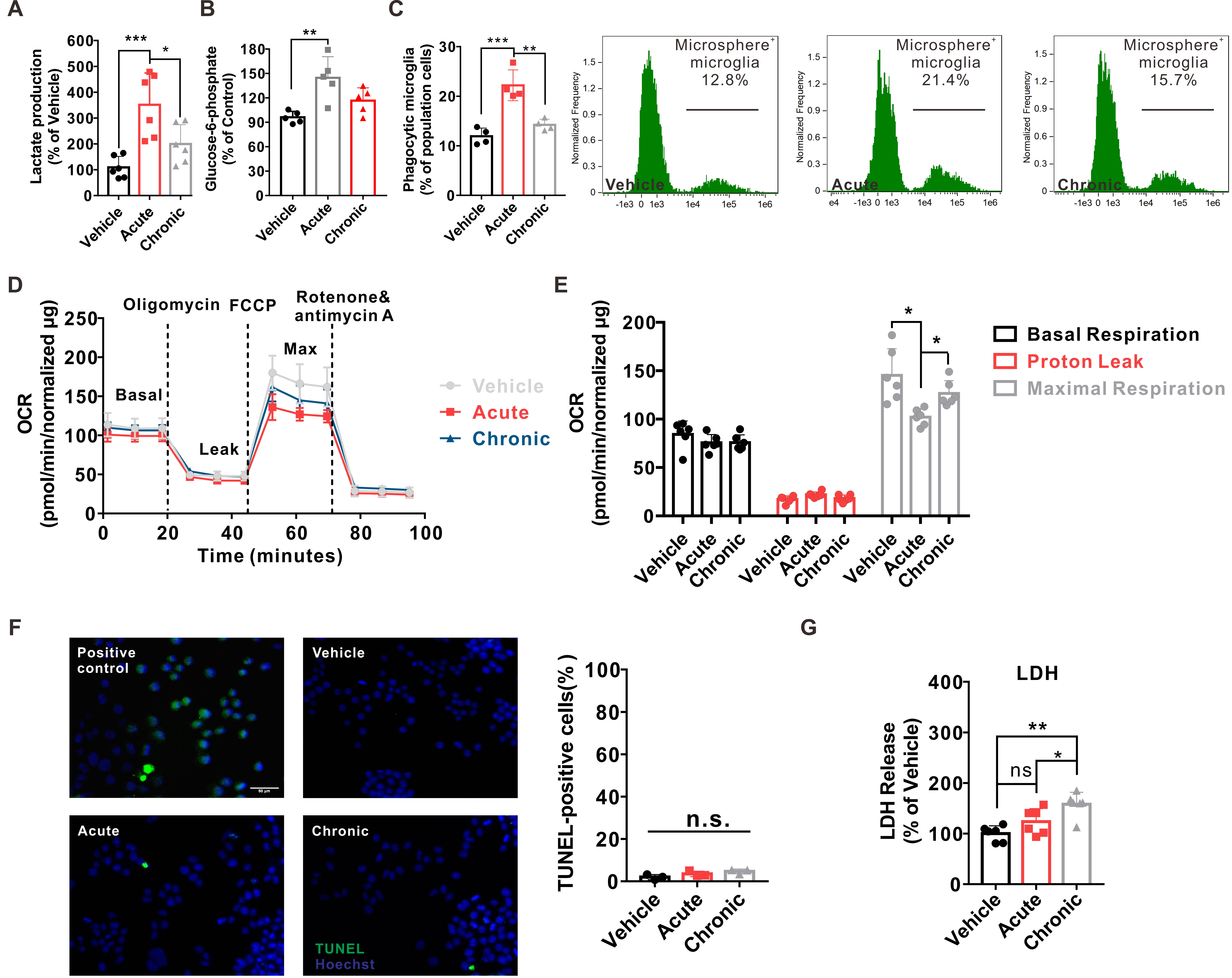

Supplement: Supplementary file 4 — Additional file 4: Figure S4. Repeated exposure of CKLF1-induced metabolic abnormalities and immune tolerance in microglia. A The level of lactate in microglia. F = 11.89. B The level of glucose-6-phosphate in microglia. F = 9.057. C Chronic stimulation of C27 led to failure of phagocytosis in microglia. The amount of zymosan phagocytosed by microglia was detected by imaging flow cytometry. F = 25.41. D Real-time changes in the oxygen consumption rate of cells. E Quantitative statistics of the basal respiration, proton leak, and maximal respiration. Basal respiration: F = 1.368; proton leak: F = 3.365; maximal respiration: F = 8.349. F Quantification of TUNEL staining induced by C27. Scale bar = 50 μm. F = 3.756. G The amount of LDH released from the microglia. F = 10.4. Data are presented as mean ± SD. *p < 0.05, **p < 0.01, ***p < 0.001 vs. vehicle or acute group. [file 12974_2023_2779_MOESM4_ESM.tif]

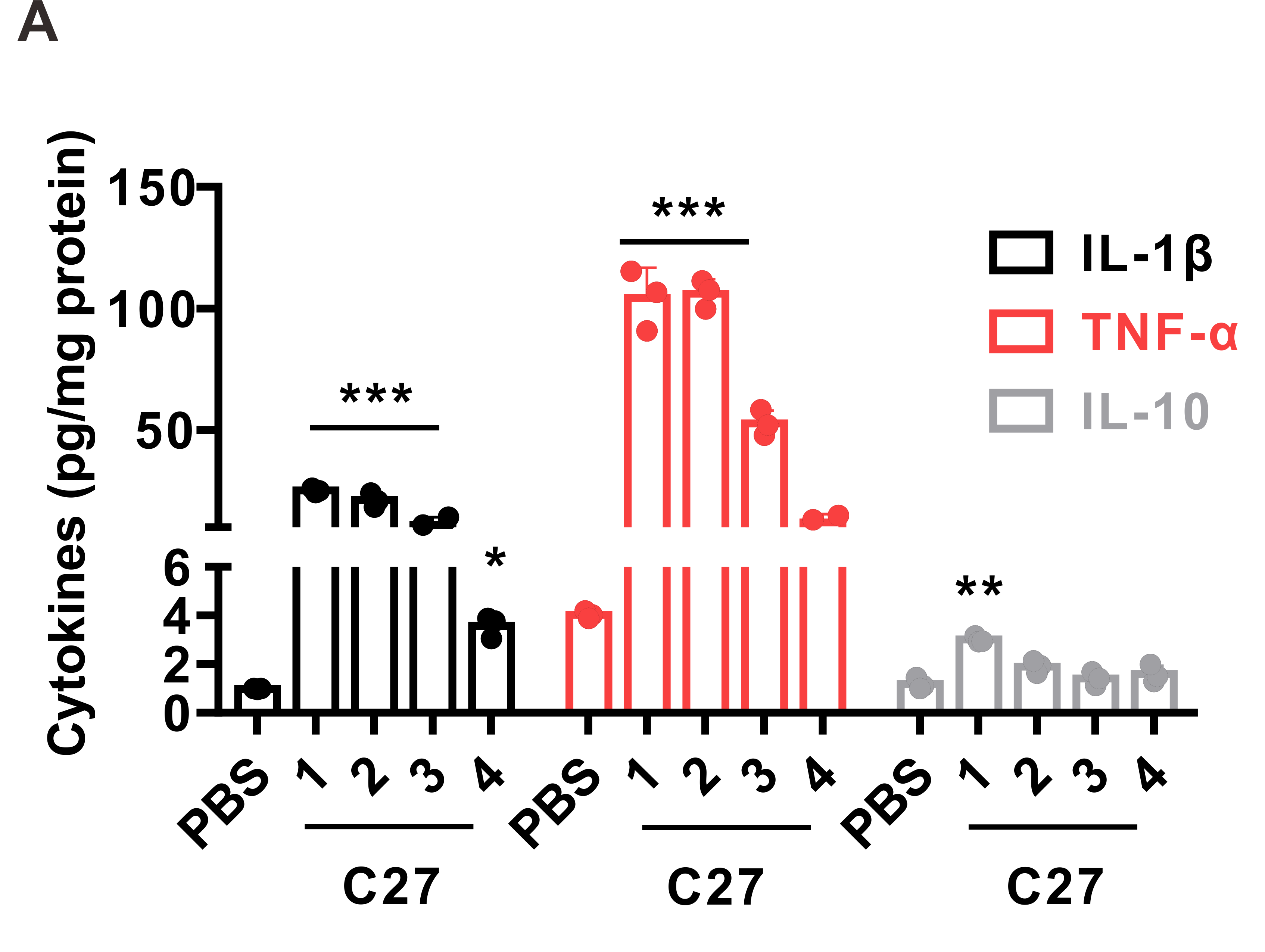

Supplement: Supplementary file 5 — Additional file 5: Figure S5. Repeated injection of C27 into M1 area of cortex reshaped the inflammation cytokines level. C27 was delivered into M1 cortex at the dose of 10 μg through stereotactic injection for different times, and the tissues was collected after 24 h of last injection. The levels of IL-1β, TNF-α and IL-10 were determined by ELISA. IL-1β: F = 89.68; TNF-α: F = 156.1; IL-10: F = 21.91. Data are presented as mean ± SD. *p < 0.05, **p < 0.01, ***p < 0.001 vs. PBS group. [file 12974_2023_2779_MOESM5_ESM.tif]
